# A high-throughput drug screen reveals means to differentiate triple-negative breast cancer

**DOI:** 10.1038/s41388-022-02429-0

**Published:** 2022-08-25

**Authors:** Milica Vulin, Charly Jehanno, Atul Sethi, Ana Luísa Correia, Milan M. S. Obradović, Joana Pinto Couto, Marie-May Coissieux, Maren Diepenbruck, Bogdan-Tiberius Preca, Katrin Volkmann, Priska Auf der Maur, Alexander Schmidt, Simone Münst, Loïc Sauteur, Michal Kloc, Marta Palafox, Adrian Britschgi, Vincent Unterreiner, Olaf Galuba, Isabelle Claerr, Sandra Lopez-Romero, Giorgio G. Galli, Daniel Baeschlin, Ryoko Okamoto, Savas D. Soysal, Robert Mechera, Walter P. Weber, Thomas Radimerski, Mohamed Bentires-Alj

**Affiliations:** 1 Department of Biomedicine, Department of Surgery, University Hospital Basel, University of Basel, Basel, Switzerland; 2 Friedrich Miescher Institute for Biomedical Research, Basel, Switzerland; 3 Swiss Institute of Bioinformatics, Basel, Switzerland; 4 Proteomics Core Facility, Biozentrum, University of Basel, Basel, Switzerland; 5 Institute of Pathology and Medical Genetics, University Hospital Basel, University of Basel, Basel, Switzerland; 6 Novartis Institutes for Biomedical Research, Basel, Switzerland; 7 Department of Surgery, University Hospital Basel, University of Basel, Basel, Switzerland; 8 Breast Cancer Center, University Hospital Basel, University of Basel, Basel, Switzerland

**Keywords:** Breast cancer, Drug screening, Differentiation, Hormone receptors

## Abstract

Plasticity delineates cancer subtypes with more or less favourable outcomes. In breast cancer, the subtype triple-negative lacks expression of major differentiation markers, e.g., estrogen receptor α (ERα), and its high cellular plasticity results in greater aggressiveness and poorer prognosis than other subtypes. Whether plasticity itself represents a potential vulnerability of cancer cells is not clear. However, we show here that cancer cell plasticity can be exploited to differentiate triple-negative breast cancer (TNBC). Using a high-throughput imaging-based reporter drug screen with 9 501 compounds, we have identified three polo-like kinase 1 (PLK1) inhibitors as major inducers of ERα protein expression and downstream activity in TNBC cells. PLK1 inhibition upregulates a cell differentiation program characterized by increased DNA damage, mitotic arrest, and ultimately cell death. Furthermore, cells surviving PLK1 inhibition have decreased tumorigenic potential, and targeting PLK1 in already established tumours reduces tumour growth both in cell line- and patient-derived xenograft models. In addition, the upregulation of genes upon PLK1 inhibition correlates with their expression in normal breast tissue and with better overall survival in breast cancer patients. Our results indicate that differentiation therapy based on PLK1 inhibition is a potential alternative strategy to treat TNBC.

## Introduction

Cellular plasticity - the ability of cells to reversibly alter their phenotype - is observed during embryonic development, in adult tissue homeostasis, upon injury, and in disease [1–5]. Cancer cells are characterized by high cellular plasticity, a hallmark that allows escape from terminal differentiation, results in aggressive disease, and in resistance to targeted therapies [6–11]. However, it is not known whether the plasticity of cancer cells, as their underlying characteristic, may at the same time make them vulnerable and be exploitable for therapy.

Breast cancer is the leading cause of cancer-related deaths in women [12]. Triple-negative breast cancer (TNBC) is a subtype of breast cancer characterized by high cellular plasticity, a high grade and low differentiation, causing high mortality [13–15].

Estrogen receptor α (ERα) belongs to the nuclear receptor family and is a key transcriptional regulator of mammary gland development and differentiation as well as breast cancer biology [16–18]. In the normal mammary gland, ERα is expressed in 40% of luminal cells that comprise the inner layer of the mammary epithelium and are surrounded by basal cells [19]. Luminal cells expressing ERα are terminally differentiated and non-proliferative [20]. However, ERα may also evoke an oncogenic, proliferative signalling pathway in ERα-positive breast tumorigenesis. This pathway can be targeted with highly effective endocrine therapies [21, 22]. The opposing effects of ERα, non-proliferative in the normal breast and proliferative in breast cancer, are marked by vastly different transcriptional outputs [23].

Here we asked whether the high cellular plasticity of TNBC can be reversed by increasing endogenous ERα expression leading to cell differentiation. As enhanced expression of ERα mRNA and protein is normally associated with DNA demethylation, we investigated mechanisms inducing ERα that are independent of ERα gene promoter demethylation. To this end, we used a high-throughput imaging-based reporter drug screen in TNBC cells with an unmethylated ERα gene promoter and identified three polo-like kinase 1 (PLK1) inhibitors as major inducers of ERα protein abundance and downstream activity. We found that PLK1 inhibition drives a cell differentiation program that leads to DNA damage, mitotic arrest, and ultimately cell death. These data suggest PLK1 as a druggable target for differentiation therapy in TNBC.

## Results

### High-throughput drug screen identifies inducers of ERα signalling in triple-negative breast cancer

**Fig. 1:**
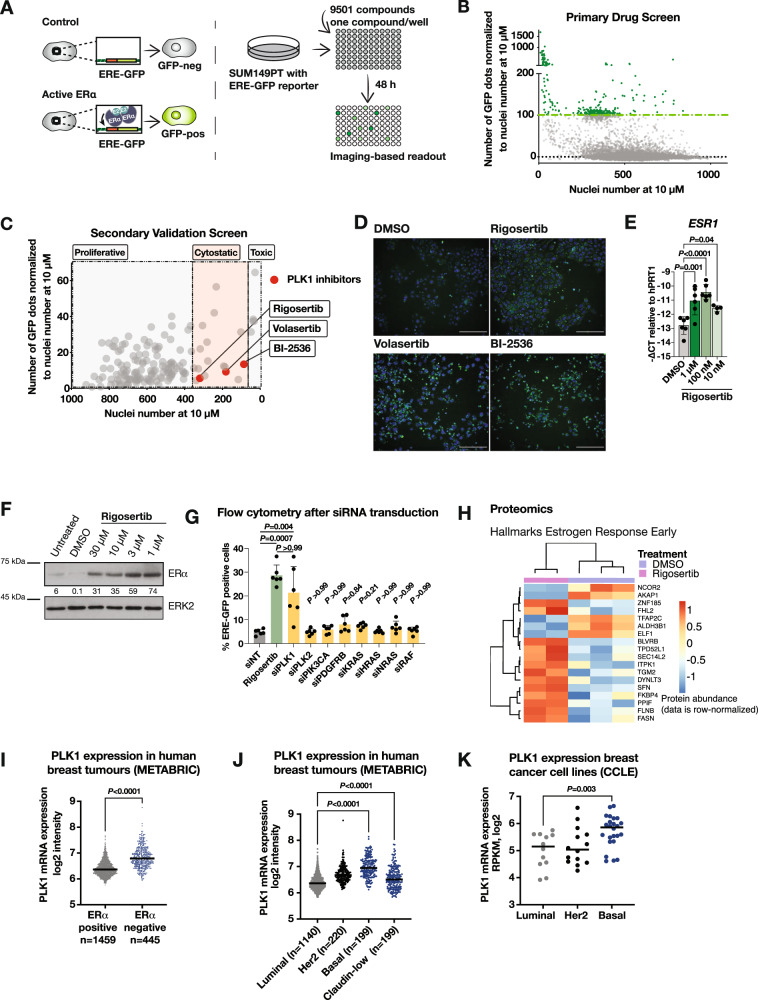
High-throughput drug screen reveals estrogen receptor α (ERα) induction in triple-negative breast cancer (TNBC) upon polo-like kinase 1 (PLK1) inhibition. **A** Schematic of the high-throughput drug screen to identify inhibitors that induce ERα signalling in TNBC. Cells without active ERα signalling do not express GFP, whereas cells with active ERα signalling trigger the ERE-GFP reporter and express GFP. Compounds were added for 48 h to SUM149PT ERE-GFP cells. GFP signal and Hoechst signal were measured with fluorescence microscopy in living cells. **B** Dot plot depicting GFP signal and nuclei number derived from Hoechst staining from the primary drug screen. **C** Dot plot depicting GFP signal and nuclei number derived from Hoechst staining from the secondary validation screen. Each point represents the mean of three technical replicates. Hits were classified as proliferative, cytostatic or toxic. PLK1 inhibitors are depicted in red. **D** Representative fluorescence microscopy live-cell images from the validation screen shown in Fig. 1C. SUM149PT cells were treated with the indicated PLK1 inhibitors for 48 h. The ERE-GFP signal is depicted in green, the Hoechst nuclei stain in blue. Scale bars: 100 µm. **E** Immunoblot showing levels of ERα and ERK2 (loading control) in SUM149PT cells treated for 72 h with rigosertib or DMSO at the indicated concentrations. **F** Bar graph representing average mRNA expression of *ESR1* in SUM149PT cells treated for 72 h with rigosertib or DMSO. *n* = 2–3 experimental replicates with 2 technical replicates each. Ordinary one-way ANOVA with multiple comparisons. Data are means ± SD. **G** Bar graph representing flow-cytometry analysis of ERE-GFP positive cells after rigosertib treatment or transfection with indicated siRNA for 72 h. *n* = 6 experimental replicates. Kruskal-Wallis test. Data are means ± SD. **H** Heatmap depicting early estrogen response proteins (from Molecular Signatures Database [MSigDB] hallmark gene sets) changing significantly upon rigosertib treatment (*n* = 2 experimental replicates) compared to DMSO (*n* = 3 experimental replicates). Data is row-normalized. **I** Dot plot showing PLK1 expression in ERα positive versus ERα negative breast cancer samples in the METABRIC [26, 27] cohort. Unpaired Student’s *t*-test. **J** Dot plot depicting PLK1 expression in different breast cancer subtypes in the METABRIC [26, 27] cohort. Ordinary one-way ANOVA with multiple comparisons. **K** Dot plot depicting PLK1 expression in different breast cancer cell lines from the Cancer Cell Line Encyclopedia (CCLE) [82]. Unpaired Student’s *t*-test.

To identify agents that induce ERα signalling in TNBC independently of ERα gene promoter demethylation, we designed a high-throughput reporter drug screen using the TNBC cell line SUM149PT. We first confirmed that SUM149PT cells are unmethylated at the ERα gene promoter as previously described (Fig. S1A) [24]. We then engineered SUM149PT cells to express an estrogen-response-element- (ERE-) green-fluorescent protein (GFP) reporter that identifies active ERα signalling. Once ERα is expressed and activated by estradiol, it binds to ERE-DNA elements and activates the transcription of GFP (Figs. 1A and S1B). Using the ERE-GFP reporter in SUM149PT cells, we conducted a high-throughput drug screen with 9 501 compounds (Supplementary Table 1).

For the primary drug screen, SUM149PT ERE-GFP cells were treated with 9 501 compounds for 48 h at a concentration of 10 µM. Subsequently, living cells were imaged by fluorescence microscopy and images were processed with an automated image-processing pipeline to quantify the GFP signal normalized to the nuclei number per compound. We identified 312 compounds that induced GFP expression above a set threshold (Fig. 1B). After individual image analysis, we recognised 131 toxic compounds, 32 artefacts and 149 hits. Toxic compounds were not selected for validation, because dead cells display high GFP autofluorescence. The 149 identified hits (1.6% of screened compounds) with the ability to induce an ERE-GFP signal in TNBC cells were selected for a secondary validation screen in which SUM149PT ERE-GFP cells were treated across eight drug concentrations with the selected compounds. Induction of ERE-GFP was concentration-dependent (Fig. S2A, B) with high reproducibility between different replicates on different plates (Fig. S2A). Compounds were classified as proliferative, cytostatic or toxic (Fig. 1C). Among the cytostatic hits, we identified three PLK1 inhibitors, namely rigosertib, volasertib and BI-2536 (Fig. 1C, D). Because PLK1 was the only target identified with more than one compound among the total hits of the drug screen, we selected these three PLK1 inhibitors for further validation studies.

### Polo-like kinase 1 (PLK1) inhibitors induce endogenous ERα signalling in triple-negative breast cancer

To validate the findings from the drug screens, we tested ERα abundance and downstream activity upon PLK1 inhibition. We found elevated *ESR1* mRNA (Figs. 1E, S3C) and ERα protein levels (Figs. 1F and S3A, B) upon rigosertib and volasertib treatments. Moreover, we confirmed an increased GFP signal stemming from the ERE-GFP reporter upon rigosertib and volasertib treatments by flow cytometry (Fig. S3D). Conversely, rigosertib treatment in SUM159PT and MDA-MB-231 cell lines that contain a methylated ERα gene promoter (Fig. S1A, [24, 25]) show no increase in *ESR1* expression, indicating that PLK1 inhibition only enhances ERα signalling in models with an unmethylated ERα gene promoter (Fig. S3E). To exclude compound off-target effects, we downregulated PLK1 and several other targets of rigosertib by siRNA and found increased ERE-GFP expression only upon knockdown of PLK1 in SUM149PT cells (Fig. 1G). To assess whether increased ERα protein abundance resulted in ERα downstream activity, we measured mRNA expression of several canonical ERα transcriptional targets by Q-PCR. We found elevated mRNA levels of *FOXA1*, *GATA3*, *AREG*, *RUNX1* and *GRHL2* upon rigosertib treatment, indicating active ERα signalling (Fig. S3F). Induction of ERα target genes was found in estradiol-free medium and did not further increase upon estradiol addition (Fig. S3F), indicating that active ERα signalling is independent of estradiol. Furthermore, estradiol did not induce proliferation in rigosertib-treated cells (Fig. S3G). Consistently, treatment with the ERα targeting therapy 4-hydroxytamoxifen (4OHT) did not decrease proliferation of SUM149PT with induced ERα signalling (Fig. S3G), indicating that the cells do not become dependent on the ERα signalling pathway. These data when combined indicate that inhibition of PLK1 evokes non-proliferative ERα signalling.

To further validate activation of ERα signalling in the cells, we analysed the global proteome after rigosertib treatment. We found proteins corresponding to the hallmark gene sets (Molecular Signatures Database [MSigDB]) for estrogen response both early and late to be overexpressed upon rigosertib treatment (Figs. 1H and S3H). This corroborates downstream activity of the ERα signalling pathway. To evaluate the relevance of our findings in breast cancer patient samples, we mined the expression of PLK1 from the METABRIC dataset [26, 27] and observed that PLK1 is mostly expressed in patients with ERα negative and basal/claudin-low tumours (Fig. 1I, J). Furthermore, the expression of PLK1 was higher in basal breast cancer cell lines than in luminal cell lines (Fig. 1K). These data indicate that PLK1 and ERα expression are anticorrelated in breast cancer and pinpoint PLK1 as an attractive target in TNBC.

To test whether the PLK1 inhibitor rigosertib binds ERα directly, we performed a dose response assay in the presence or absence of 4OHT in the SUM149PT cell line. We found no difference in cell numbers in response to rigosertib in the presence or absence of 4OHT, indicating that rigosertib does not compete with 4OHT in binding ERα (Fig. S4A). Next, to further evaluate whether rigosertib can bind ERα, we treated the ERα-positive cell line T47D ERE-luciferase cells short-term (8 h) with rigosertib and measured luciferase activity, a well-known assay to assess estrogenic effects in cells. We observed no increase in luciferase activity after short-term rigosertib treatment, further indicating that rigosertib has no estrogenic effects (Fig. S4B). Finally, we treated both the ERα-positive cell line MCF7 wild-type and the *ESR1* mutant cells (D538G and Y537S) that no longer respond to 4OHT with rigosertib. Treatment with rigosertib decreased the number of *ESR1* mutant MCF7 cells, suggesting that it acts independently of ERα binding (Fig. S4C). In summary, PLK1 inhibition increases ERα signalling independently of estradiol, yet PLK1 inhibitors do not bind ERα directly.

To assess if the increased ERα downstream signalling is a direct consequence of increased ERα levels, we treated SUM149PT cells engineered to express a short hairpin targeting ERα (shERα) with rigosertib. This has prevented the increased expression of ERα downstream targets seen upon rigosertib treatment (Fig. S4D). Of note, rigosertib still decreased the number of shERα cells, consistent with the known effect of PLK1 on cell cycle progression (Fig. S4E) [28]. Taken together, these data indicate that PLK1 inhibition induces estradiol-independent non-proliferative ERα signalling in TNBC cells.

### PLK1 inhibition upregulates cell differentiation programs

**Fig. 2:**
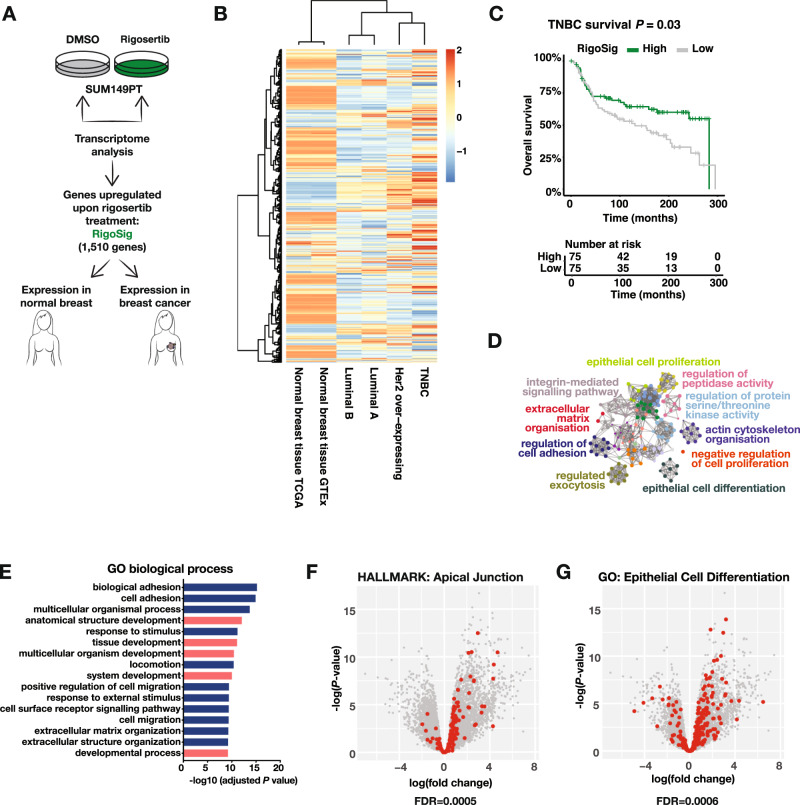
PLK1 inhibition upregulates cell differentiation programmes. **A** Schematic outlining the generation of the RigoSig gene set (upregulated genes upon rigosertib treatment, 1 510 genes, cut off: adjusted *P*-value < 0.01 and log fold change >1, *n* = 3 experimental replicates, Supplementary Table 2) and the comparison of breast cancer patient samples with normal breast samples. **B** Expression heatmap of RigoSig genes. Data shown for breast cancer subtypes and normal breast samples measured by the TCGA consortium [29] and normal breast samples measured by the GTEx consortium [30]. Data is row-normalized. TNBC: triple-negative breast cancer. **C** Kaplan–Meier curve of RigoSig genes in TNBC samples (from METABRIC [26, 27]) showing increased overall survival upon high expression of RigoSig genes. Samples in the top and bottom quartile of signature expression are compared. **D** Pathway enrichment analysis (Metascape) of RigoSig gene set. **E** Pathway enrichment analysis (Gene Ontology biological process) of RigoSig gene set. Developmental pathways are shown in red. Volcano plot for rigosertib versus DMSO treatment contrast. Geneset enrichment FDR are calculated using MROAST. Genes shown in red belong to genesets (**F**) Apical Junction (MSigDB hallmark gene sets), and (**G**) Epithelial Cell Differentiation (Gene Ontology).

To characterize induced endogenous ERα signalling in the context of TNBC, we performed global transcriptomic profiling in SUM149PT cells treated with rigosertib or a DMSO control (Fig. 2A). We identified a set of 1 510 genes whose expression was significantly upregulated upon rigosertib treatment that we named the “RigoSig gene set” (Supplementary Table 2). We first compared expression of RigoSig genes in patient samples from different breast cancer subtypes in the TCGA cohort [29] with normal breast tissue from two different consortia, GTEx [30] and TCGA [29]. The RigoSig gene set correlated more with normal breast tissue than with luminal-like breast cancer, indicating active, non-oncogenic ERα signalling (Fig. 2B). Furthermore, high expression of RigoSig genes in the METABRIC cohort of TNBC patients [26, 27] predicted better overall survival (Fig. 2C).

Pathway enrichment analysis of RigoSig genes revealed that epithelial cell differentiation, cell adhesion and integrin signalling were among the most significantly enriched molecular functions after rigosertib treatment (Fig. 2D). RigoSig genes were associated with developmental pathways, namely anatomical structure development (GO:0048856), tissue development (GO:0009888), multicellular organism development (GO:0007275), system development (GO:0048731) and developmental process (GO:0032502) (Fig. 2E). Gene set enrichment analysis (GSEA) revealed enrichment of apical junction (hallmarks), epithelial cell differentiation (GO:0030855), and estrogen response early and late (hallmarks) (Figs. 2F, G, and S5A). To investigate whether the similarity between PLK1 inhibited cells and normal epithelial tissue results from a common cell cycle arrest, we subtracted cell cycle genes from the RigoSig gene set and repeated the GSEA. Epithelial cell differentiation was significantly enriched in the modified RigoSig gene set even without taking cell cycle genes into account, indicating that the observed differentiation phenotype is independent of the cell cycle arrest (Fig. S5B). Furthermore, we compared enrichment of known ERα transcriptional targets specific to normal breast or breast cancer [23] in the RigoSig gene set and found ERα targets from normal breast to be significantly enriched upon PLK1 inhibition, while ERα breast cancer targets were not (Fig. S5C). Altogether, the data indicate that PLK1 inhibitor-evoked ERα signalling in the context of TNBC resembles normal rather than oncogenic ERα signalling and thus illustrates increased cellular differentiation.

### PLK1 inhibition induces DNA damage with subsequent mitotic arrest and cell death

**Fig. 3:**
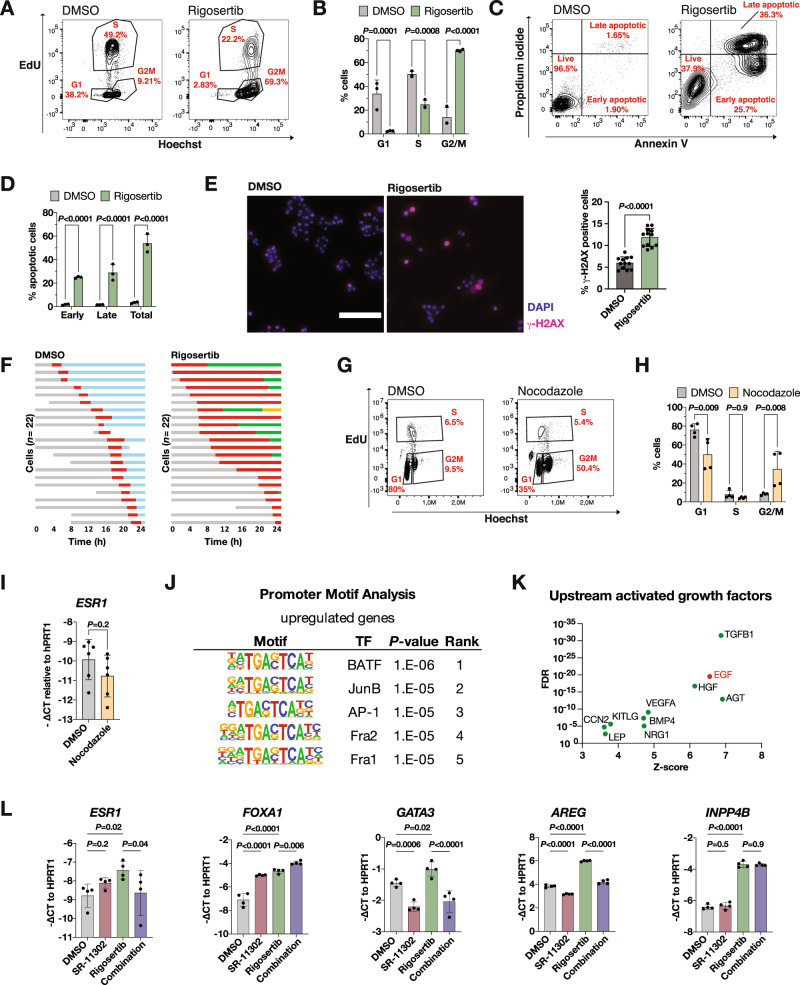
PLK1 inhibition induces DNA damage with subsequent mitotic arrest. **A** Representative flow-cytometry dot plots of EdU/Hoechst cell cycle staining of SUM149PT cells treated for 3 days with 100 nM rigosertib or DMSO. **B** Bar graph depicting the proportion of cells in different cell cycle states based on EdU/Hoechst cell cycle staining shown in Fig. 3A. *n* = 3 experimental replicates. Ordinary two-way ANOVA with multiple comparisons. Data are means ± SD. **C** Representative flow-cytometry dot plots of propidium iodide/annexin V staining of SUM149PT cells treated for 3 days with 100 nM rigosertib or DMSO. **D** Bar graph depicting the proportion of cells in different apoptotic states based on the propidium iodide/annexin V staining shown in Fig. 3C. *n* = 3 experimental replicates. Ordinary two-way ANOVA with multiple comparisons. Data are means ± SD. **E** Left panel: Representative fluorescence microscopy images of SUM149PT cells treated for 48 h with 100 nM rigosertib or DMSO and stained with γ-H2AX. The γ-H2AX signal is depicted in magenta, the DAPI nuclei stain in blue. Scale bar: 200 µm. Right panel: Bar graph showing the percentage of γ-H2AX positive cells. *n* = 2 experimental replicates with 4 technical replicates each. Mann-Whitney U-test. Data are means ± SD. **F** Bar graphs representing cell cycle states of individual cells tracked over time with time-lapse microscopy. Each horizontal bar represents one cell. Gray: interphase; red: mitosis (from DNA condensation to anaphase or mitotic slippage); blue: interphase after mitosis; green: interphase after mitotic slippage (DNA decondensation without division); yellow: cell death. **G** Representative flow-cytometry dot plots of EdU/Hoechst cell cycle staining of SUM149PT cells treated for 3 days with 1 µM nocodazole or DMSO. **H** Bar graph depicting the proportion of cells in different cell cycle states based on EdU/Hoechst cell cycle staining shown in Fig. 3E. *n* = 4 experimental replicates. Ordinary two-way ANOVA with multiple comparisons. Data are means ± SD. **I** Bar graphs representing average mRNA expression of *ESR1* in SUM149PT cells treated with 1 µM nocodazole or DMSO. *n* = 3 experimental replicates with 2 technical replicates each. Unpaired Student’s *t*-test. Data are means ± SD. **J** Table depicting promoter motif enrichment of the RigoSig gene set. Top-5 enriched motifs are depicted. TF, transcription factor. **K** Dot plot depicting the growth factor upstream regulators identified by Ingenuity Pathway Analysis of the RigoSig gene set. **L** Bar graphs representing average mRNA expression of *ESR1* and downstream targets in SUM149PT cells treated for 72 h with 100 nM rigosertib, 10 µM SR-11302, a combination treatment of rigosertib and SR-11302 or DMSO. *n* = 2 experimental replicates with 2 technical replicates each. Ordinary two-way ANOVA with multiple comparisons. Data are means ± SD.

To investigate the underlying determinants of the increased cell differentiation observed, we assessed phenotypic changes induced by PLK1 inhibition. Cell cycle analysis showed that cells treated with the PLK1 inhibitor rigosertib are arrested mostly in G2/M consistent with the known effects of PLK1 during the cell cycle (Fig. 3A, B) [28]. In addition, we found an increase in early and late apoptotic cells upon rigosertib treatment compared to the DMSO control (Fig. 3C, D), providing evidence that PLK1 inhibition induces mitotic arrest and subsequent cell death. Time-lapse microscopy of individually tracked cells revealed that rigosertib-treated cells showed prolonged mitosis with subsequent mitotic slippage (Fig. 3F). Given that cell cycle arrest at G2/M often indicates the presence of unrepaired double-stranded breaks in mitosis, we investigated whether rigosertib affects DNA damage in SUM149PT cells. Rigosertib-treated cells displayed increased γ-H2AX staining intensity compared to DMSO control counterparts, suggesting that the mitotic arrest observed upon PLK1 inhibition stemmed from increased DNA damage (Fig. 3E).

The strong cell cycle arrest upon PLK1 inhibition prompted us to investigate if ERα expression could be induced by other G2/M cell cycle inhibitors. Treatment of SUM149PT cells with nocodazole induced G2/M cell cycle arrest (Fig. 3G, H), but in contrast to PLK1 inhibition, we found no upregulation of *ESR1* expression, indicating that the upregulation of ERα signalling is specific to PLK1 inhibition (Fig. 3I).

These findings led us to investigate mechanistically how PLK1 inhibition in TNBC cells converges to induce ERα signalling, cell differentiation and DNA damage. We examined the motifs in the promoter region of RigoSig genes and found increased activity of five members of the AP-1 transcription factor family (BATF, JunB, AP-1, Fra2, Fra1) as the top transcription factors controlling upregulated genes upon rigosertib treatment (Fig. 3J). This finding along with the fact that AP-1 can act as a co-factor of ERα in an estradiol independent way [31] could explain why PLK1 inhibition restores endogenous ERα signalling in TNBC independently of estradiol. ERα/AP1 interactions require growth factors like EGF or IGF1 [31, 32]. Hence, to investigate the upstream activated growth factors in the RigoSig genes, we conducted an Ingenuity Pathway Analysis and found an upregulated EGF signature among the top two most upregulated signatures, suggesting that ERα/AP1 interactions are responsible for the upregulation of ERα targets (Fig. 3K). To assess if the increase in ERα signalling is functionally dependent on AP-1, we treated the cells with an AP-1 inhibitor (SR-11302) in addition to rigosertib. We observed that the increase of some (*ESR1, GATA3, AREG*), but not all, ERα targets can be prevented by inhibiting AP-1 (Fig. 3L). These data indicate that ERα/AP1 interactions are partially responsible for the estradiol-independent activity of ERα signalling upon PLK1 inhibition. In summary, we show that PLK1 inhibition induces DNA damage and subsequent mitotic arrest and cell death, and that the induction of ERα signalling is partially mediated by ERα/AP-1 interactions.

### PLK1 inhibition induces a sustained change in cell fate

**Fig. 4:**
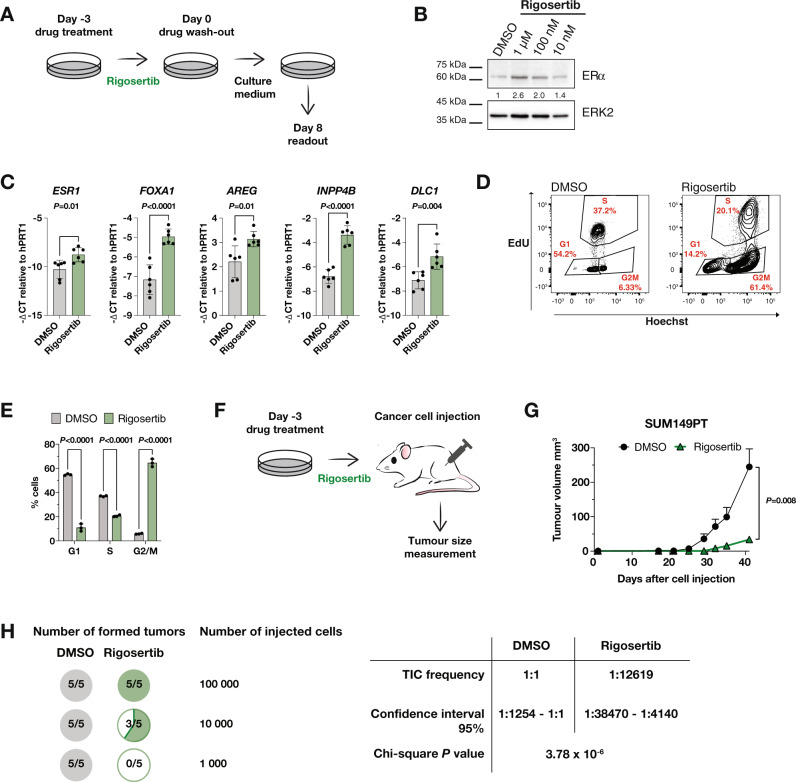
PLK1 inhibition induces a sustained change in cell fate. **A** Schematic of the drug wash-out experiment. SUM149PT cells were treated for 3 days with rigosertib or DMSO. Subsequently, the drug was washed out and cells were cultured for eight more days without the drug and then harvested for downstream experiments. **B** Immunoblot showing levels of ERα and ERK2 (loading control) in SUM149PT cells treated with the indicated concentrations of rigosertib as depicted in Fig. 4A. **C** Bar graphs representing average mRNA expression of *ESR1* and downstream targets in SUM149PT cells treated with 100 nM rigosertib or DMSO as depicted in Fig. 4A. *n* = 2 experimental replicates with 2 technical replicates each. Unpaired Student’s *t*-test. Data are means ± SD. **D** Representative flow-cytometry dot plots of EdU/Hoechst cell cycle staining of SUM149PT cells treated with 100 nM rigosertib or DMSO as depicted in Fig. 4A. **E** Bar graph depicting the proportion of cells in different cell cycle states based on EdU/Hoechst cell cycle staining shown in Fig. 4D. *n* = 3 experimental replicates. Ordinary two-way ANOVA with multiple comparisons. Data are means ± SD. **F** Schematic of the experimental setup for in vitro treated SUM149PT cells grown as mouse xenografts in NSG mice. Cells were treated for 3 days with 1 µM rigosertib or DMSO prior to injection. **G** Kinetics of primary tumour growth of SUM149PT cells treated in vitro with 1 µM rigosertib (*n* = 5 mice) or DMSO (*n* = 5 mice) as depicted in Fig. 4F. Mann–Whitney U-test. Data are means ± SD. **H** Left panel: Pie charts depicting quantification of tumour incidence upon in vitro treatment of SUM149PT cells as in Fig. 4F. Right panel: Table summarizing the frequency of tumour initiating cells (TICs) and respective statistical analysis. Chi-square test.

To investigate whether PLK1 inhibition induces a durable change in cell fate, we treated SUM149PT cells with rigosertib and examined the cellular phenotype after drug wash-out (Fig. 4A). Protein and mRNA analyses revealed upregulated ERα levels eight days post rigosertib treatment, as well as elevated ERα transcriptional targets of normal breast [23] (Fig. 4B, C). In addition, the vast majority of cells treated with rigosertib were still arrested in G2/M phase at that time, as indicated by a 10-fold increase in the G2/M phase population and a decrease in the S and G0/G1 phase populations of 2- and 5-fold, respectively, compared to the DMSO control counterparts (Fig. 4D, E).

Having shown that PLK1 inhibition leads to sustained accumulation of ERα protein and increased cell differentiation in TNBC, we investigated its potential to decrease tumorigenesis in vivo. To this end, we treated SUM149PT cells in vitro with rigosertib and subsequently injected 1 × 10^6^ viable cells into the mammary fat pads of immunocompromised NOD scid gamma (NSG) mice (Fig. 4F). We found that cells injected after PLK1 inhibition were growth-impaired in vivo, indicating decreased tumorigenic potential (Fig. 4G). Limiting dilution experiment where we injected different numbers of in vitro rigosertib treated cells into the mammary fat pads of NSG mice show that rigosertib treated cells were growth-impaired, as evidenced by a decreased tumour initiating cell (TIC) frequency (Fig. 4H). Altogether, the data indicate that sustained differentiation upon PLK1 inhibition decreases tumorigenesis of TNBC cells.

### PLK1 inhibition decreases tumour growth in vivo

**Fig. 5:**
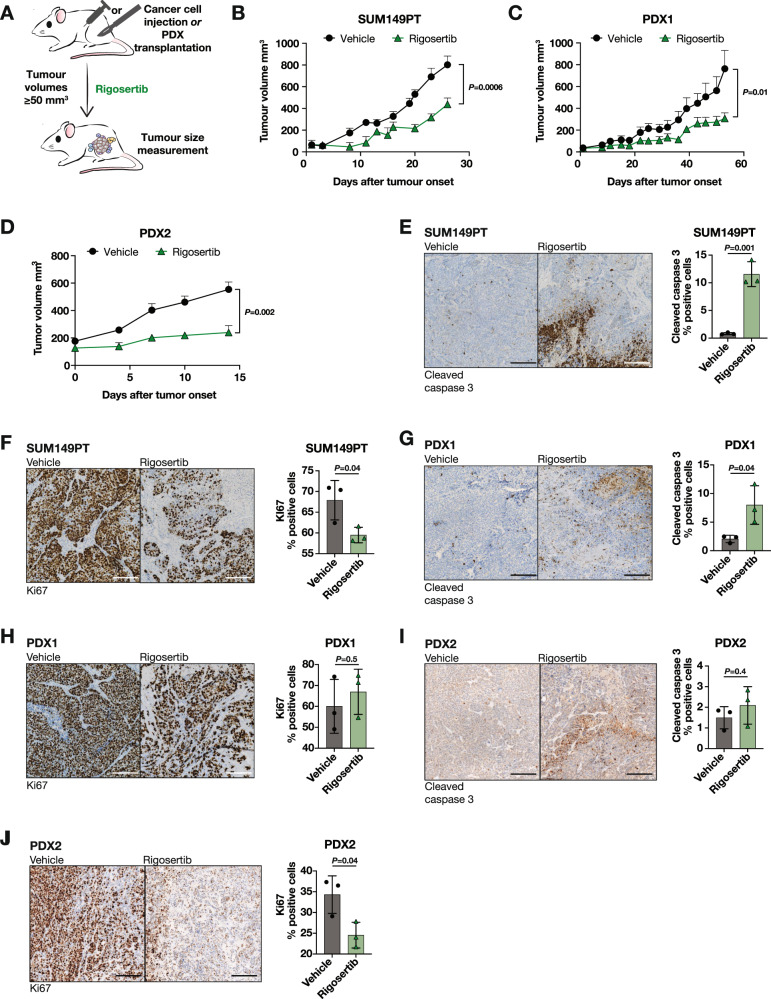
PLK1 inhibition reduces tumour growth in vivo. **A** Schematic depicting the experimental setup for in vivo treatment of SUM149PT or PDX1 mouse xenografts. Rigosertib or vehicle treatments were started once the tumours reached a volume ≥50 mm^3^. **B** Kinetics of primary tumour growth of SUM149PT cells treated in vivo with rigosertib (*n* = 10 mice) or vehicle (*n* = 9 mice) as depicted in Fig. 5A. Mann–Whitney U-test. Data are means ± SEM. **C** Kinetics of primary tumour growth of PDX1 cells treated in vivo with rigosertib (*n* = 5 mice) or vehicle (*n* = 6 mice) as depicted in Fig. 5A. Mann–Whitney U-test. Data are means ± SEM. **D** Kinetics of primary tumour growth of PDX2 cells treated in vivo with rigosertib (*n* = 6 mice) or vehicle (*n* = 11 mice) as in Fig. 5A. Mann–Whitney U-test. Data are means ± SEM. **E** Representative images of cleaved caspase 3 staining (left panel) and bar graph quantification of cleaved caspase 3 positive cells (right panel) in tissue sections of SUM149PT tumours treated with rigosertib or vehicle. Scale bars: 300 µm. *n* = 3 tumours per group. Unpaired Student’s *t*-test. Data are means ± SD. **F** Representative images of Ki67 (left panel) and bar graph quantification of Ki67 positive cells (right panel) in tissue sections of SUM149PT tumours treated with rigosertib or vehicle. Scale bars: 300 µm. *n* = 3 tumours per group. Unpaired Student’s *t*-test. Data are means ± SD. **G** Representative images of cleaved caspase 3 staining (left panel) and bar graph quantification of cleaved caspase 3 positive cells (right panel) in tissue sections of PDX1 tumours treated with rigosertib or vehicle. Scale bars: 300 µm. *n* = 3 tumours per group. Unpaired Student’s t-test. Data are means ± SD. **H** Representative images of Ki67 (left panel) and bar graph quantification of Ki67 positive cells (right panel) in tissue sections of PDX1 tumours treated with rigosertib or vehicle. Scale bars: 300 µm. *n* = 3 tumours per group. Unpaired Student’s *t*-test. Data are means ± SD. **I** Representative images of cleaved caspase 3 staining (left panel) and bar graph quantification of cleaved caspase 3 positive cells (right panel) in tissue sections of PDX2 tumours treated with rigosertib or vehicle. Scale bars: 300 µm. *n* = 3 tumours per group. Unpaired Student’s *t*-test. Data are means ± SD. **J** Representative images of Ki67 (left panel) and bar graph quantification of Ki67 positive cells (right panel) in tissue sections of PDX2 tumours treated with rigosertib or vehicle. Scale bars: 300 µm. *n* = 3 tumours per group. Unpaired Student’s *t*-test. Data are means ± SD.

To assess the effects of PLK1 inhibition on already-formed tumours, we injected SUM149PT cells or transplanted patient-derived xenografts (PDX) models into the mammary fat pads of NSG mice. We then treated animals with rigosertib or vehicle once the tumours were established (>50 mm^3^) (Fig. 5A). In all models, we found that rigosertib significantly delayed tumour growth (Fig. 5B–D).

To investigate whether the observed decrease in tumour volume was due to cell cycle arrest and/or increased cell death, we stained the tumours for the proliferation marker Ki67 and the apoptosis marker cleaved caspase 3. In SUM149PT cells, cleaved caspase 3 levels were increased and Ki67 levels were decreased upon rigosertib treatment (Fig. 5E, F). Among the PDX models, PDX1 tumours showed increased cleaved caspase 3, while PDX2 tumours showed decreased levels of Ki67 (Fig. 5G–J). We found no increase in ERα expression upon in vivo treatment with rigosertib, possibly because ERα positive cells halt proliferation providing a selective advantage to ERα negative cells in the tumours (Fig. S6). These data indicate that depending on the model and its growth kinetics, the decrease in tumour growth is either mediated by increased cell death and/or decreased proliferation.

## Discussion

Cancer cell phenotypic plasticity is central to tumour initiation, metastasis and resistance to therapy and poses a major obstacle for the cure of cancer [6–9, 11]. Cancer cell plasticity was recently added to the hallmarks of cancer, allowing malignant cells to escape from terminal differentiation [10]. Among breast cancer subtypes, TNBC displays high cellular plasticity and dedifferentiation, making it a prime disease model to study plasticity in cancer [13, 14]. Whether cell plasticity is also a potential vulnerability that can be reversed is not clear. Here we show that targeting PLK1 in TNBC induces endogenous ERα signalling, which is widely accepted as a marker of differentiation in the breast.

ERα is a key transcription factor controlling differentiation of luminal cells in the mammary gland [16, 17]. During ERα-positive breast tumorigenesis, ERα signalling enhances proliferation, is oncogenic, and can be targeted with endocrine therapies [21, 22]. Here, we report that PLK1 inhibitors induce endogenous ERα signalling in TNBC, resembling normal rather than oncogenic ERα signalling. PLK1 inhibition does not reprogram TNBC cells towards dependency on an oncogenic ERα signalling pathway and does not restore susceptibility to endocrine treatment, it rather differentiates TNBC cells towards normal breast cells. Therefore, increasing the expression of ERα in an environment that is foreign to the transcription factor is insufficient to reprogram the cells towards ERα dependency. Indeed, the absence of hormone-dependent activity might be explained by the lack of ERα transcriptional co-factors or by an epigenetic landscape in which classical ERα-dependent enhancers are not accessible. Here, we uncovered that ERα downstream targets are increased upon PLK1 inhibition, independently of estradiol. Furthermore, we find that the increase of some, but not all, ERα targets can be prevented by AP-1 inhibition. Estradiol independent ERα signalling has been described for a variety of ERα-cofactors such as AP1, CREB1 [33], and XBP-1 [34]. Even though AP-1 motifs are highly enriched in our RigoSig gene set, it is conceivable that additional ERα-partners contribute to the increased ERα signalling pathway.

Other studies have uncovered that TNBC cells with a methylated ERα gene promoter can be targeted with DNA-methylation inhibitors or histone deacetylase inhibitors to induce ERα expression [25, 35–42]. Furthermore, MAPK inhibition was shown to induce ERα signalling [24]. Of note, among the 149 hits of our drug screen, we found several pan-kinase inhibitors indicating that inhibition of other kinases might contribute to increased ERα expression in TNBC. In previous studies, induced ERα expression sensitized TNBC cells marginally to endocrine treatment [24, 43–45]. However, none of the previous studies reported increased differentiation following induced ERα signalling in TNBC. Conceivably, the induction of endogenous ERα signalling upon PLK1 inhibition is accompanied by DNA damage, which was previously shown to induce differentiation in other tumour models [46–48], as well as in normal keratinocytes [49]. This indicates a conserved homeostatic effect of DNA damage in triggering differentiation. Further studies investigating the effects of DNA damage-inducing agents on ERα signalling in TNBC are warranted.

We found no increase of ERα upon in vivo treatment with rigosertib, possibly because ERα-evoked differentiation halts cell proliferation providing a selective advantage to ERα negative cells in the tumours (Fig. S6). Alternatively, there might be additional mechanisms at play in vivo that downregulate ERα signalling.

While differentiation therapy has been widely successful in the treatment of acute promyelocytic leukaemia (APL) [50], its usefulness in the treatment of solid tumours is controversial [51]. A notable example comes from a recent study demonstrating that cellular plasticity could be exploited to trans-differentiate breast cancer cells into functional adipocytes [52]. Although theoretically attractive, differentiation therapy is challenged by the heterogeneity of solid tumours that harbour multiple oncogenic cooperating pathways [51]. In this context, targeting PLK1 is an appealing alternative as it is a key regulator of the cell cycle and its activity is often altered in cancer [28]. Despite being an essential gene, the effect of PLK1 is multi-layered. A link between PLK1 and cell fate was recently shown in the skin, where inactivation of PLK1 in mouse squamous epithelia induced full differentiation [53]. The authors proposed a differentiation-mitosis checkpoint, where upon prolonged cell cycle arrest cells differentiate avoiding apoptosis [53]. It remains unknown if this checkpoint is conserved across different cell types [53–56].

Finally, growing experimental evidence suggests that cancer cell plasticity and dedifferentiation drive immune evasion [57]. Therefore, differentiation therapy could ultimately increase susceptibility of cancer cells to immunotherapies [58] that are approved in the treatment of TNBC [59].

In summary, we show that PLK1 inhibition induces ERα signalling in TNBC. ERα signalling is a mark of increased differentiation, identifying non-proliferative cells with low tumorigenic potential. We thus pinpoint PLK1 as a targetable factor for differentiation therapy in TNBC.

## Materials and methods

### Cell lines

The SUM149PT cell line was kindly provided by Dr. Julie Lang (Cleveland, Ohio, USA), while the SUM159PT cell line was kindly provided by Dr. Charlotte Kupperwasser (Boston, Massachusetts, USA). Both cell lines are commercially available (Asterand, Detroit, MI, USA). The SUM149PT and the SUM159PT cell lines were cultured in Ham’s F12 (Sigma, cat#N6658) with 5% fetal calf serum (FCS) (Sigma, cat# F7524), 5 μg/mL human recombinant insulin (Sigma, cat# 91077 C), 1 μg/mL hydrocortisone (Sigma, cat# H0888), and 1×penicillin/streptomycin (Sigma, cat# P4333). The T47D, the MCF7 and the MDA-MB-231 cell lines were from ATCC and cultured according to their protocol. The MCF7 mutant cell lines were kindly provided by Dr. Ben Ho Park (Nashville, Tennessee, USA) [60]. Cell line identities were confirmed by short tandem repeat (STR) sequencing and all cell lines were routinely tested for mycoplasma contamination.

### In vivo establishment of a primary human breast cancer xenograft

Primary human breast cancer xenograft 1 (PDX1) was established as previously described [61]. After obtaining written informed consent from the patient allowing the usage of their tissue for scientific research purposes, primary breast cancer tissue from a triple-negative breast cancer patient was surgically resected and directly transplanted into the mammary fat pads of female NSG (NOD-*scid-Il2rg*^null^) mice. The tissue was obtained at the University Hospital Basel in the project ID: 2018–00729 that was approved by the Swiss authorities (EKNZ, Ethics Committee northwest/central Switzerland) in compliance with the Declaration of Helsinki. PDX2 was previously described [61, 62].

### Animal experiments

All mouse experiments were performed in accordance with the Swiss animal welfare ordinance and approved by the cantonal veterinary office of Basel Stadt. Female NSG mouse colonies were maintained in the animal facility of the Department of Biomedicine of the University of Basel in accordance with Swiss guidelines on animal experimentation. Mice were kept in a pathogen-free environment with a controlled light cycle from 6:00 h to 18:00 h, a temperature of 20 to 23 °C and humidity of 50 to 60%. Mice were allowed to acclimatize for a minimum of 7 days before each experiment.

For orthotopic engraftment of breast cancer cell lines, 1 × 10^6^ SUM149PT cells were resuspended in 100 µL matrigel (Corning, cat# 356237) and PBS (Gibco, cat# 20012-019) mixed (1:1) and injected into the fourth mammary fat pads of 8 to 12-week-old female NSG mice. For experiments with PDX1, tumours were also transplanted into the fourth mammary fat pads of 8 to 12-week-old female NSG mice. Mice were supplemented with estradiol for the whole duration of the experiments, either by implanting estradiol pellets (Belma Technologies, cat# E2M/90) or by supplementing the drinking water with 8 µg/mL estradiol (Sigma, cat# E8875; stock diluted in ethanol) as previously described [63, 64]. Tumour diameters were measured with manual callipers and tumour volumes calculated by the formula 0.5 × [(larger diameter) × (smaller diameter)^2^]. When tumours reached a volume >50 mm^3^, mice were randomized into two treatment groups, rigosertib or vehicle. For in vivo treatments, rigosertib (Lubio Science, cat# S1362) was first dissolved in polyethylene glycol (Fluka, cat# 25322-68-3) and further diluted with PBS to a concentration of 22 mg/mL. Mice received 200 µL intraperitoneal injections of rigosertib or vehicle (1:1 PEG to PBS) twice a week, which corresponds to 200 mg/kg. Mice were euthanized before the maximal tumour volumes permitted by the cantonal veterinary office of Basel Stadt (1 500 mm^3^) were reached.

### Tumorigenic potential

To assess tumorigenic potential of cells after PLK1 inhibition, SUM149PT cells were treated in vitro for 3 days with rigosertib or DMSO in estradiol-free cell culture medium. Subsequently, 1 × 10^6^ SUM149PT cells were resuspended in 100 µL matrigel (Corning, cat# 356237) and PBS (Gibco, cat# 20012-019) mixed (1:1) and injected into the fourth mammary fat pads of 8 to 12-week-old female NSG mice. Mice were supplemented with estradiol for the whole duration of the experiments in the drinking water, with 8 µg/mL estradiol (Sigma, cat# E8875; stock diluted in ethanol) as previously described [63, 64]. For the limiting dilution experiment, mice were not supplemented with estradiol. SUM149PT cells were treated in vitro for 3 days with rigosertib or DMSO in estradiol-free cell culture medium. Subsequently, 100 000, 10 000 or 1 000 SUM149PT cells were resuspended in 100 µL matrigel and PBS mixed (1:1) and injected into the fourth mammary fat pads of 8 to 12-week-old female NSG mice. The frequencies of TICs were calculated and statistically compared using the Extreme Limiting Dilution Analysis (ELDA) online tool [65]. Tumour volumes were monitored as described above.

### DNA methylation analysis

Genomic DNA was converted by bisulfite treatment using the EpiTect Bisulfite Kit (Qiagen, cat# 59104) and subsequently subjected to methylation-specific PCR using the Epitect MSP kit (Qiagen, cat# 59305). Two regions of the *ESR1* promoter mostly associated with ERα protein expression were amplified by methylation-specific PCR: ER 1 and ER 5 [66, 67]. For each region, a primer pair for methylated and unmethylated DNA, respectively, were used, as described previously [67]. PCR products were purified in a 2% agarose gel and subsequently submitted to Sanger sequencing.

### Drug screen

We screened 9501 compounds belonging to two compound libraries, the Mechanism-of-Action Box (MoA Box) [68] and the Novartis core screening set for external collaboration. Cells were counted with a Cedex HiRes Cell Counter (Innovatis) and 2000 cells per well in 50 µL standard cell culture medium were plated with a Multidrop 384 (Thermo Electron Corporation, Thermo Scientific) on poly-D lysine coated 384 well plates (Corning, cat# 7244). The plates were centrifuged for 10 s at 400 rpm and left for 20 min at RT before being placed into incubators at 37 °C and 95% humidity. On the following day, the medium was changed to fresh medium containing 10 nM estradiol (Sigma, cat# E8875) and 100 ng/mL Hoechst 33342 (Thermo Molecular Probes, cat#H3570) with a CyBio SELMA (Analytik Jena AG). The compounds dissolved in 90% DMSO were transferred from plates containing compounds (Novartis) to the plates containing cells, with an Echo 555 (Labcyte) in 45 min intervals. After 48 h of incubation, images were captured with a CV7000 (Yokogawa) confocal microscope (20X, NA = 0.45) equipped with a sCMOS camera X3 (pixel size 6.5 µm). The images were analysed with the Yokogawa Analysis Software (YAS: Yokogawa). The nuclei (Hoechst) and the GFP dots were identified and counted in each image. The output of the image analysis was a well mean of the number of GFP dots divided by the number of nuclei. For the primary drug screen, cells were treated at a concentration of 10 µM. For the secondary drug screen, cells were treated across eight drug concentrations ranging from 10 µM to 3.16 nM.

### Cell proliferation assay

For proliferation assays, cells were plated on day 0 in 96 well plates (5 000 cells per well) and allowed to adhere overnight. For in vitro experiments, rigosertib (Lubio Science, Cat# S1362) was dissolved in DMSO, while 4-hydroxytamoxifen (4OHT) (Sigma, cat# H7904) and estradiol (Sigma, cat# E8875) were dissolved in ethanol. On day 1, the culture medium was changed to estradiol-free cell culture medium. Estradiol-free cell culture medium is composed of phenol red-free Ham’s F12 (Caisson Labs, cat# HFL05) supplemented with charcoal-stripped 2% FCS (Gibco, cat# A3382101), 5 μg/mL human recombinant insulin (Sigma, cat# 91077 C), 1 μg/mL hydrocortisone (Sigma, cat# H0888), and 1× penicillin/streptomycin (Sigma, cat# P4333). Rigosertib or DMSO were added on day 1 at the indicated concentrations. On day 3, 10 nM estradiol, 4OHT (at the indicated concentrations) or ethanol were added to the cells, and refreshed on day 6. On day 9, the medium was removed and cells were fixed and stained with sulforhodamine B (Sigma, cat# 230162) as previously described [69]. In brief, cells were fixed with cold 3.3% trichloroacetic acid (Sigma, cat# T6399) at 4 °C for 1 h or overnight. Plates were washed with slow-running tap water and air-dried at RT. Subsequently, 100 µL of 0.057% sulforhodamine B solution were added to each well and plates were left at RT for 30 min. The plates were then washed with 1% acetic acid and air-dried at RT. The protein-bound dye was solubilized with 200 µL 10 mM Tris base solution (pH 10.5) and the optical density (OD) measured at 510 nm using the Synergy H1 microplate reader (BioTek). For the dose response assay, cells were treated for 3 days with 100 nM rigosertib in combination with 1 µM 4OHT or ethanol and subsequently fixed and stained with sulforhodamine B as described above.

### Lentiviral infections

Lentiviruses were produced either by PEI transfection of 293 T cells as previously described [70] or by co-transfection of 293 T cells with X-tremeGENE 9 DNA transfection reagent (Roche, cat# 06365787001) and DNA (X-tremeGene9: DNA ratio was 2.5:1). The titre of each lentiviral batch was determined in SUM149PT, SUM159PT and T47D cells. For lentiviral infections, cells were spin infected (1200 × g for 45 min at 32 °C) in the presence of 8 µg/mL hexadimethrine bromide (Sigma, cat# H9268) and incubated overnight. Infections were performed at a multiplicity of infection (MOI) of 0.5 viral particles per cell. The ERE-GFP vector was purchased from BioCat: pGreenFire-Estrogen Response Element with EF1-hygro (cat# CS920A-1-SBI). The *ESR1* plasmid was a gift from Richard D. Iggo (Bordeaux, France) and described previously [71].

### Transient gene silencing

siRNAs were ordered as ON-TARGET plus SMART pools (Dharmacon). The siRNA IDs were as follows: siNT (D-001810-10-05), siPLK1 (L-003290-00-0005), siPLK2 (L-003325-00-0005), siROS1 (L-003173-00-0005 5), siPDGFRB (L-003163-00-0005 5), siPIK3CA (L-003018-00-0005 5), siKRAS (L-005069-00-0005 5), siNRAS (L-003919-00-0005 5), siHRAS (L-004142-00-0005 5), and siRAF1 (L-003601-00-0005 5). Transfection of siRNAs was performed according to the manufacturer’s protocol with DharmaFECT 1 (Dharmacon, cat# T-2001-02) as transfection reagent.

### ERE-GFP detection by flow cytometry

Cell lines were grown in standard cell culture medium with or without inhibitors and siRNAs as indicated, and supplemented with 10 nM estradiol for 3 days. Estradiol was refreshed after 2 days. Subsequently, cells were detached using trypsin-EDTA, resuspended in growth medium and counted. Cells were washed with PBS, strained over 40 µm filters and resuspended in PBS with 1% FCS. DAPI (0.2%, Invitrogen, cat# D1306) was added to exclude dead cells. Single cells were gated on the basis of their forward and side-scatter profiles and pulse width was used to exclude doublets. Cells were analysed on a BD LSR Fortessa Cell analyser (BD Biosciences) using the BD FACS Diva Software (BD Biosciences, v.7). Results were analysed with the FlowJo software (v.5).

### Cell cycle staining

Cells were treated for the indicated times with 100 nM rigosertib or DMSO in estradiol-free cell culture medium. Cells were labelled for 2 h with 10 µM EdU. Detection of EdU was conducted using the Click-iT EdU Alexa Fluor™ 647 assay kit (Invitrogen, cat# C10419) according to the manufacturer’s guidelines. DNA content was stained with Hoechst 33342 (Invitrogen, cat# H3570) and cells analysed on a BD LSR Fortessa Cell analyser (BD Biosciences) using the BD FACS Diva Software (BD Biosciences, v.7). Results were analysed with the FlowJo software (v.5).

### Annexin V staining

Cells were treated for 3 days with 100 nM rigosertib or DMSO in standard cell culture medium. Subsequently, cells were washed twice with cold cell staining-buffer (BioLegend, cat# 420401) and resuspended in Annexin V binding buffer (BioLegend, cat# 422201) at a concentration of 1 × 10^6^cells/mL. Aliquots of 50 µL of the cell suspension were transferred to test tubes and 2.5 µL of Annexin V Alexa Fluor 647 (BioLegend, cat# 640943) and 5 µL of propidium iodide solution (BioLegend, cat# 421301) were added. Cells were gently vortexed and incubated for 15 min at RT in the dark. Finally, 200 µL of Annexin V binding buffer (BioLegend, cat# 422201) was added to each sample and cells were analysed on a BD LSR Fortessa Cell analyser (BD Biosciences) using the BD FACS Diva Software (BD Biosciences, v.7). Results were analysed with the FlowJo software (v.5).

### Protein lysate preparation and immunoblotting

For ERα immunoblots, cells were grown in estradiol-free cell culture medium and treated with inhibitors for the indicated times. Cells lysates for immunoblotting were prepared in whole-cell lysis buffer (150 mM NaCl [Merck, cat# 1.06404.5000], 10% glycerol [Sigma, cat# G6279], 1% NP 40 [Fluka, cat# 74385], 0.5% sodium deoxycholate [Sigma, cat# 30970], 2 mM EDTA [Gerbu, cat# 1034], 0.1% SDS [Sigma, cat# L3771], and 20 mM Tris-HCl, pH 8 [AppliChem, cat# A1086]) supplemented freshly with 1x protease inhibitor cocktail (Complete EDTA-free, Roche, cat# 11836153001), 1x phosphatase inhibitor cocktail (Sigma, cat# P0044), 0.2 mM sodium orthovanadate (Sigma, cat# 450243) and 20 mM sodium fluoride (Merck, cat# 1.06449.0250), followed by sonication (10 cycles of 30 s ON and 30 s OFF) using a Bioruptor Pico device (Diagenode). Protein lysates (30–60 µg) were subjected to SDS–PAGE, transferred to PVDF membranes (Immobilon-P, Sigma, cat# IPVH85R) and blocked for 1 h at RT with 5% milk in TBS/0.05% Tween 20. Membranes were incubated either overnight or for 40 h at 4 °C with primary antibodies and exposed to secondary HRP-coupled anti-mouse (Merck, cat# GENA931) or anti-rabbit (Merck, cat# GENA934) antibodies (1:5 000–10 000, GE Healthcare) either for 2 h at RT or overnight at 4 °C. Membranes were developed using WesternSure Chemiluminescent Substrate (Li-cor Biosciences, cat# 926-95000) or WesternBright Sirius HRP substrate (Advansta, cat# K-12043-C20). The following antibodies were used: anti-ERK2 (1:2 000, Santa Cruz, cat# sc-1647), anti-ERα (1:250, Thermo Fisher Scientific, cat# MA5-14501). Pixel densities of respective bands on blots were quantified using ImageJ (FIJI, v.2.3) [72] and normalized to ERK2. Results are representative of at least three different experiments.

### Histology and immunohistochemistry

All tissues were fixed in 4% paraformaldehyde (PFA):PBS solution for 24 h at RT. Samples were then dehydrated, embedded in paraffin and sectioned (3–4 µm) with a Microm HM 340E (Thermo Fisher Scientific). All immunohistochemistry experiments were performed using a Ventana DiscoveryXT instrument (Roche Diagnostics) following the Research IHC DAB Map XT procedure. Counter staining was performed with hematoxylin II and bluing reagent (Ventana, Roche diagnostics).

For cleaved caspase 3 staining, slides were pre-treated with Cell Conditioning medium 1 (CC1, Roche Diagnostics) for 40 min at 95 °C, followed by 40 min of incubation with blocking buffer (1X Casein, Surmodics, cat# PBSC-0100-01). Cleaved caspase 3 primary antibody (Cell Signaling, cat# 9661, 1:100 in blocking buffer) was incubated for 1 h at 37 °C, followed by secondary antibody incubation (polymer-HRP anti-rabbit, cat# Nichirei, 414142 F) for 1 h at 37 °C, and revealed by the Discovery ChromoMap DAB detection kit.

For Ki67 staining, slides were pre-treated with CC1 for 64 min at 95 °C, followed by 40 min of incubation with blocking buffer (1X Casein). Ki67 primary antibody (Thermo Fischer Scientific, cat# MA5-14520, 1:50 in blocking buffer) was incubated for 1 h at 37 °C, followed by secondary antibody incubation (polymer-HRP anti-rabbit) for 1 h at 37 °C, and revealed by the Discovery ChromoMap DAB detection kit.

For ERα staining, slides were pre-treated with CC1 for 72 min at 95 °C, followed by 8 min of incubation with blocking buffer (1X Casein). ERα primary antibody (Thermo Fisher Scientific, cat# MA5-14501, 1:50 in blocking buffer) was incubated for 1 h at 37 °C, followed by secondary antibody incubation (polymer-HRP anti-rabbit) for 1 h at 37 °C, and revealed by the Ventana Amplification kit (cat# 760-080).

Whole sections were scanned using a NanoZoomer S60 digital slide scanner (Hamamatsu) and quantified using HALO (v3.1). Classification of tissue area and exclusion of tumour necrosis and stroma were performed using implemented software tools. Ratio of positive cells was determined using the multiplex IHC module. Representative images of histological sections were captured using a Nikon Ti2-E inverted microscope (20X, NA = 0.75; 10X, NA = 0.45 and 4X, NA = 0.2 objectives) equipped with a Nikon DS-Ri2 camera.

### γ-H2AX staining

For γ-H2AX staining, cells were grown for 48 h with 100 nM rigosertib or DMSO in standard cell culture medium. Subsequently, cells were fixed with 4% paraformaldehyde (in PBS, Electron Microscopy Sciences, cat# 15714) for 15 min and permeabilized for 45 min with 0.15% triton X (in PBS, Merck, cat# 1086431000) at RT. Next, 2% bovine serum albumin (BSA, in PBS; Sigma, cat# 10735094001) blocking solution was added to the cells for 30 min and the cells were subsequently incubated overnight with phospho-histone H2A.X (Ser139) (γ-H2AX) antibody (Cell Signaling, cat# 2577 S) diluted 1:300 in blocking solution. On the next day, cells were washed with PBS and incubated overnight with the secondary anti-mouse antibody conjugated to Alexa Fluor 647 (Invitrogen, cat# A-21241) and DAPI (1:200, Invitrogen, cat# D1306). Cells were then washed and images captured using a Nikon Ti2-E inverted microscope (20X, NA = 0.75, 10X, NA = 0.45 and 4X, NA = 0.2 objectives) equipped with a Photometrics Prime 95B camera. Images were acquired with the Nikon NIS software and quantified using QuPath (v.0.3.0) [73]. Cell count was determined using the cell detection module. Ratio of positive cells was identified using the single measurement classifier with simple thresholding.

### Time-lapse imaging

The time-lapse movie was acquired every 5 min for 24 h as a z-stack (10x, 4 field of views (FOVs) per well, 4 wells per condition, 30 µm range, 10 µm z-distance) in mCherry and brightfield using a CQ1 (Yokogawa) confocal spinning disk microscope and Yokogawa acquisition software. Sum intensity projections of the mCherry channel were used in the Trackmate Plugin [74] of FIJI for segmentation using StarDist (Minimum Spot Quality 0.5) [75] and results were submitted to tracking using the overlap tracking with the following settings (mode: precise, min IoU 0.3, 2).

The KNIME Analytics Platform 4.4.4 was used to filter out tracks with gaps, merge events, complex points, track length of less than 4 frames, tracks with all objects with an area smaller or equal to 0.01, tracks with objects with a mean mCherry intensity of less than 200. The further analysis only included tracks with a standard deviation of the mean mCherry intensity over all frames of the track higher than 400, indicative for a condensation event.

Time points of condensation were selected based on a decrease of nuclear area and a simultaneous increase mean mCherry intensity. Time points of decondensation were selected based on an increase of nuclear area and a simultaneous decrease of mean mCherry intensity.

### mRNA isolation and Q-PCR

For *ESR1* and *ESR1* downstream targets Q-PCRs, cells were grown in estradiol-free cell culture medium and treated with inhibitors for the indicated times. Cells were treated for 8 h with estradiol, 4OHT or ethanol before mRNA extraction.

Total RNA was extracted using the RNeasy Plus Mini Kit (Qiagen, cat# 74134) according to the manufacturer’s protocol. The iScript cDNA conversion kit (Biorad, cat# 1708891) was used to transcribe 500 ng–1 µg of total RNA. For quantitative real time PCR (Q-PCR), fluorescence detection was performed using the ViiA™ 7 Real-Time PCR System (Applied Biosystems) according to the manufacturer’s protocol in a reaction volume of 10 µL containing 1x PrimeTime Gene Expression Master Mix (IDT, cat# 1055772) and 50 ng cDNA. The following probes were used: 1x IDT (Integrated DNA technologies) assays for quantification of *HPRT1* (Hs.PT.58.v.45621572), *ESR1* (Hs.PT.58.14846478), *FOXA1* (Hs.PT.58.1788586), *GATA3* (Hs.PT.584308511), *AREG* (Hs.PT.56a.38817860), *RUNX1* (Hs.PT.58.24461868), *GRHL2* (Hs.PT.58.40379174), *INPP4B (Hs.PT.58.19965063)*, and *DLC1 (Hs.PT.58.27928708)*. All measurements were performed in technical duplicates and the arithmetic mean of the Ct values was used for calculations: target gene mean Ct values were normalized to the respective house-keeping gene (*HPRT1*), mean Ct values (internal reference gene, Ct) to obtain the minus delta Ct (–ΔCt) values.

### RNA-sequencing and analysis

RNA was quality-checked on the TapeStation instrument (Agilent Technologies) using the RNA ScreenTape (Agilent, cat# 5067–5576). Library preparation was performed, starting from 200 ng total RNA, using the TruSeq Stranded mRNA Library Kit (Ilumina, cat# 20020595) and the TruSeq RNA UD Indexes (Ilumina, cat# 20022371). Fifteen cycles of PCR were performed. Quality-checking on the Fragment Analyzer (Advanced Analytical) using the Standard Sensitivity NGS Fragment Analysis Kit (Advanced Analytical, cat# DNF-473) revealed the excellent quality of the libraries (average concentration was 34 ± 4 nmol/L and average library size was 330 ± 6 base pairs). Samples were pooled to equal molarity. The pool was quantified by Fluorometry using the QuantiFluor ONE dsDNA System (Promega, cat# E4871). Libraries were sequenced Paired-End 51 bases (in addition: 8 bases for index 1 and 8 bases for index 2) using the NovaSeq 6000 instrument (Illumina) and the SP Flow-Cell loaded at a final concentration in Flow-Lane of 380 pM and including 1% PhiX. Primary data analysis was performed with the Illumina RTA version 3.4.4. A total of 1.03 billion reads passing Illumina quality control (PF reads) were collected in total for the 24 samples, i.e. 42.9 ± 4.7 million PF reads on average per sample.

Reads were aligned to the human genome (UCSC version hg38AnalysisSet) with STAR. The output was sorted and indexed with samtools. Stand-specific coverage tracks per sample were generated by tiling the genome in 20-bp windows and counting the 5’end of reads per window using the function bamCount from the bioconductor package bamsignals. These window counts were exported into bigWig format using the bioconductor package rtracklayer. The rsubread::featureCounts function was used to count the number of reads (5’ends) overlapping with the exons of each gene assuming an exon union model (gene annotation: ensembldb_Homo_sapiens_GRCh38_ensembl_96.sqlite). Differential gene expression analysis was performed using limma-voom framework. Pathway enrichment analysis was performed using gProfiler [76] and Metascape [77]. For gene set enrichment analysis (GSEA), human gene sets were obtained from (http://bioinf.wehi.edu.au/software/MSigDB) and enrichment analyses were performed using MROAST available in bioconductor package limma. Transcription factor motif analysis was performed using Homer (4.11) [78]. For the identification of upstream regulators, RigoSig genes were subjected to QIAGEN Ingenuity Pathway Analysis. Upstream regulators were clustered based on their biological group [79].

### Proteomics analysis using tandem mass tags

Sample aliquots comprising 25 µg of peptides were labelled with isobaric tandem mass tags (TMT 10-plex, Thermo Fisher Scientific). Peptides were resuspended in 20 µL labelling buffer (2 M urea, 0.2 M HEPES, pH 8.3) by sonication and 5 µL of each TMT reagent (0.8 mg in 80 µL DMSO) were added to the individual peptide samples followed by a 1 h incubation at 25 °C with shaking at 500 rpm. To control for ratio distortion during quantification, a peptide calibration mixture consisting of six digested standard proteins mixed in different amounts was added to each sample before TMT labelling (for details see Ahrné et al., 2016 [80]). To quench the labelling reaction, 1.5 µL aqueous 1.5 M hydroxylamine solution was added and samples were incubated for another 5 min at 25 °C with shaking at 500 rpm followed by pooling of all samples. The pH of the sample pool was increased to 11.9 by adding 1 M phosphate buffer (pH 12) and incubated for 20 min at 25 °C with shaking at 500 rpm to remove TMT labels linked to peptide hydroxyl groups. Subsequently, the reaction was stopped by adding 2 M hydrochloric acid until pH<2. Finally, peptide samples were further acidified using 5% TFA, desalted using Sep-Pak Vac 1cc (50 mg) C18 cartridges (Waters) according to the manufacturer’s instructions and dried under vacuum.

TMT-labelled peptides were fractionated by high-pH reversed phase separation using a XBridge Peptide BEH C18 column (3,5 µm, 130 Å, 1 mm × 150 mm, Waters) on an Agilent 1260 Infinity HPLC system. Peptides were loaded onto the column in buffer A (20 mM ammonium formate in water, pH 10) and eluted using a two-step linear gradient from 2% to 10% in 5 min and then to 50% buffer B (20 mM ammonium formate in 90% acetonitrile, pH 10) over 55 min at a flow rate of 42 µL/min. Elution of peptides was monitored with a UV detector (215 nm, 254 nm) and a total of 36 fractions were collected, pooled into 12 fractions using a post-concatenation strategy as previously described [81] and dried under vacuum. Dried peptides were resuspended in 0.1% aqueous formic acid and subjected to LC–MS/MS analysis using a Q Exactive HF Mass Spectrometer fitted with an EASY-nLC 1000 (both Thermo Fisher Scientific) and a custom-made column heater set to 60 °C. Peptides were resolved using a RP-HPLC column (75 μm × 30 cm) packed in-house with C18 resin (ReproSil-Pur C18–AQ, 1.9 μm resin; Dr. Maisch GmbH) at a flow rate of 0.2 μL/min. The following gradient was used for peptide separation: from 5% B to 15% B over 10 min to 30% B over 60 min to 45 % B over 20 min to 95% B over 2 min followed by 18 min at 95% B. Buffer A was 0.1% formic acid in water and buffer B was 80% acetonitrile, 0.1% formic acid in water. The mass spectrometer was operated in DDA mode with a total cycle time of approximately 1 s. Each MS1 scan was followed by high-collision-dissociation (HCD) of the ten most abundant precursor ions with dynamic exclusion set to 30 s. For MS1, 3e6 ions were accumulated in the Orbitrap over a maximum time of 100 ms and scanned at a resolution of 120000 FWHM (at 200 m/z). MS2 scans were acquired at a target setting of 1e5 ions, maximum accumulation time of 100 ms and a resolution of 30000 FWHM (at 200 m/z). Singly charged ions and ions with unassigned charge state were excluded from triggering MS2 events. The normalized collision energy was set to 35%, the mass isolation window was set to 1.1 m/z and one microscan was acquired for each spectrum.

The acquired raw files were analysed using the SpectroMine software (Biognosys AG, 1.0.20235.13.16424). Spectra were searched against a human database consisting of 20 404 protein sequences (downloaded from Uniprot on 2019/03/07) and 392 commonly observed contaminants. Standard Pulsar search settings for TMT10 (“TMT_Quantification”) were used and resulting identifications and corresponding quantitative values were exported on the PSM level using the “Export Report” function. Acquired reporter ion intensities in the experiments were employed for automated quantification and statistical analysis using our in-house developed SafeQuant R script (v2.3) [80]. This analysis included adjustment of reporter ion intensities, global data normalization by equalizing the total reporter ion intensity across all channels, summation of reporter ion intensities per protein and channel, calculation of protein abundance ratios and testing for differential abundance using empirical Bayes moderated t-statistics. Finally, the calculated *P*-values were corrected for multiple testing using the Benjamini-Hochberg method.

Differential abundance analysis of proteomics data was performed using limma-voom framework. Human gene sets were obtained from (http://bioinf.wehi.edu.au/software/MSigDB) and enrichment analyses were performed using MROAST available in bioconductor package limma.

### Statistical analysis

Cell line groups and animals of the same age were randomized based on standard laboratory practice procedures. The investigators were not blinded to allocation during experiments and outcome assessment. Values represent the means ± SD or the means ± SEM for in vivo treatments, as indicated in the figure legends. Depending on the type of experiment, data were tested for normal distribution and analysed using ordinary one-way ANOVA, ordinary two-way ANOVA, the Kruskal–Wallis test, the Mann-Whitney U-test or the unpaired Student’s *t*-test as indicated in the figure legends. Groups with similar variance were compared using parametric statistical tests, otherwise groups were compared using nonparametric statistical test. No sample-size calculations were performed. Sample size was determined to be adequate based on the magnitude and consistency of measurable differences between groups. Experimental replicates are independent experiments. Technical replicates are tests or assays run on the same sample multiple times.

## Supplementary information

Supplementary figure and table legends

Supplementary figure 1

Supplementary figure 2

Supplementary figure 3

Supplementary figure 4

Supplementary figure 5

Supplementary figure 6

Supplementary table 1

Supplementary table 2

## Data Availability

The mRNA-sequencing data were deposited in the GEO database with the accession number GSE184295. The proteomics data were deposited in the PRIDE database with the accession code PXD028495.
