# Supplementary material for: A high-throughput drug screen reveals means to differentiate triple-negative breast cancer
Source: Oncogene. 2022 Aug 25;41(39):4459–73. doi: 10.1038/s41388-022-02429-0 (PMC9507968; doi:10.1038/s41388-022-02429-0)
Supplement: Supplementary file 1 — Supplementary figure and table legends [file 41388_2022_2429_MOESM1_ESM.docx]

**Supplementary figure legends**

**Figure S1. SUM149PT are mostly unmethylated at the ERα gene promoter. A**, DNA methylation profile of two *ESR1* CpG islands mostly associated with ERα protein expression of one ERα positive cell line (T47D) and two ERα negative cell lines (SUM159PT and SUM149PT). **B**, Representative flow-cytometry dot plot of ERE-GFP signal from one ERα positive cell line (T47D) and two TNBC cell lines (SUM159PT and SUM149PT), each transduced additionally with *ESR1*.

**Figure S2. Induction of ERα signalling is dose-dependent. A**, Heatmap of GFP signal normalized to nuclei count from the secondary validation screen of the individual screening plates. **B**, Dose response curves of ERE-GFP signal normalized to nuclei count upon different drug concentrations extracted from the secondary validation screen of three PLK1 inhibitors (rigosertib, volasertib, BI-2536). Each point represents the mean of three experimental replicates.

**Figure S3. PLK1 inhibition induces endogenous ERα protein and downstream signalling independently of estradiol (E2). A**, Immunoblot showing levels of ERα and ERK2 (loading control) in SUM149PT cells treated for 72 h with rigosertib or DMSO at the indicated concentrations. **B**, Immunoblot showing levels of ERα and ERK2 (loading control) in SUM149PT cells treated for 72 h with volasertib or DMSO at the indicated concentrations. **C**, Bar graph representing average mRNA expression of *ESR1* in SUM149PT cells treated for 72 h with volasertib or DMSO. *n =* 3 experimental replicates with 2 technical replicates each. Ordinary one-way ANOVA with multiple comparisons. Data are means ± SD. **D**, Bar graph showing flow-cytometry analysis of ERE-GFP positive cells after rigosertib or volasertib treatments for 72 h. *n* =  3 experimental replicates. Kruskal-Wallis test. Data are means ± SD. **E**, Bar graphs representing average mRNA expression of *ESR1* in SUM159PT and MDA-MB-231 cells treated for 72 h with rigosertib or DMSO. *n =* 3 experimental replicates with 2 technical replicates each. Unpaired Student’s t-test. Data are means ± SD. **F**, Bar graphs representing average mRNA expression of *ESR1* and downstream targets in SUM149PT cells treated for 72 h with rigosertib or DMSO. *n =* 3 experimental replicates with 2 technical replicates each. Ordinary two-way ANOVA with multiple comparisons. Data are means ± SD. **G**, Bar graph representing absorbance at 510 nm of SUM149PT stained cells, treated with the indicated concentrations of rigosertib/DMSO or 4-hydroxytamoxifen (4OHT)/ethanol (EtOH) with or without estradiol (E2). *n =* 3 experimental replicates with 2 technical replicates each. Ordinary two-way ANOVA with multiple comparisons. Data are means ± SD. **H**, Heatmap depicting late estrogen response proteins (from Molecular Signatures Database [MSigDB] hallmark gene sets) changing significantly upon rigosertib treatment (*n*= 2 experimental replicates) compared to DMSO (*n*= 3 experimental replicates). Data is row-normalized.

**Figure S4. PLK1 inhibitors do not compete with 4OHT to bind ERα directly. A**, Dot plot depicting the normalized dose response of nuclei count upon rigosertib treatment with or without 4-hydroxytamoxifen (4OHT). *n*= 2 experimental replicates. Data are means ± SD. **B**, Bar graphs representing average luciferase signal in SUM149PT cells treated for 8 h with 1 µM rigosertib, 10 nM estradiol or DMSO. *n =* 3 experimental replicates with 3 technical replicates each. Kruskal-Wallis test. Data are means ± SD. **C**, Bar graph representing absorbance at 510 nm of stained MCF7 wild-type or *ESR1* mutant cells, treated with the indicated concentrations of rigosertib or DMSO. n =  3 experimental replicates with 2 technical replicates each. Ordinary two-way ANOVA with multiple comparisons. Data are means ± SD. **D**, Bar graphs representing average mRNA expression of *ESR1* and downstream targets in SUM149PT shNT or shERα cells treated for 72 h with rigosertib or DMSO. *n =* 3 experimental replicates with 2 technical replicates each. Ordinary two-way ANOVA with multiple comparisons. Data are means ± SD. **E**, Bar graph representing absorbance at 510 nm of stained SUM149PT shNT or shERα cells, treated with the indicated concentrations of rigosertib or DMSO. n =  3 experimental replicates with 2 technical replicates each. Ordinary two-way ANOVA with multiple comparisons. Data are means ± SD.

**Figure S5. PLK1 inhibition upregulates non-oncogenic ERα signalling pathways. A**, Volcano plots for rigosertib versus DMSO treatment contrast. Left panel: Genes from the MSigDB hallmark gene sets Estrogen Response Early are shown in red. Right panel: Genes from the MSigDB hallmark gene sets Estrogen Response Late are shown in red. FDR calculations are derived from enrichment analyses using MROAST. **B**, Volcano plot for rigosertib versus DMSO treatment contrast without cell cycle genes. Genes shown in red belong to the geneset Epithelial Cell Differentiation (Gene Ontology). FDR calculation is derived from enrichment analyses using MROAST. **B**, Volcano plot for rigosertib versus DMSO treatment contrast. Left panel: ERα target genes in the normal mammary gland [23] are shown in red. Right panel: ERα target genes in breast cancer [23] are shown in red. FDR calculations are derived from enrichment analyses using MROAST. **C**, Pathway enrichment analysis (KEGG pathways) of RigoSig gene set. Estrogen signalling pathway is shown in red.

**Figure S6. ERα protein is not detected upon PLK1 inhibition *in vivo*.** Upper panel: Representative images of ERα staining in tissue sections of SUM149PT tumours (upper panel) or PDX1 tumours (middle panel) treated with rigosertib or vehicle. Scale bars: 300 µm. Lower panel: Representative image of ERα staining in a human ERα positive tumour used as a positive control for the ERα staining. Scale bar: 300 µm.

**Supplementary table legends**

**Supplementary Table 1.** List of compounds screened during the primary drug screen and obtained screening results. N indicates the GFP signal stemming from the ERE-GFP reporter and Ch 2 indicates the number of nuclei of SUM149PT ERE-GFP cells upon treatment with the indicated inhibitor.

**Supplementary Table 2.** List of genes upregulated upon rigosertib treatment. Cut offs are adjusted *P-*value < 0.01 and log fold change >1.
