# Supplementary figures and images for: A high-throughput drug screen reveals means to differentiate triple-negative breast cancer
Source: Oncogene. 2022 Aug 25;41(39):4459–73. doi: 10.1038/s41388-022-02429-0 (PMC9507968; doi:10.1038/s41388-022-02429-0)

Figure S1

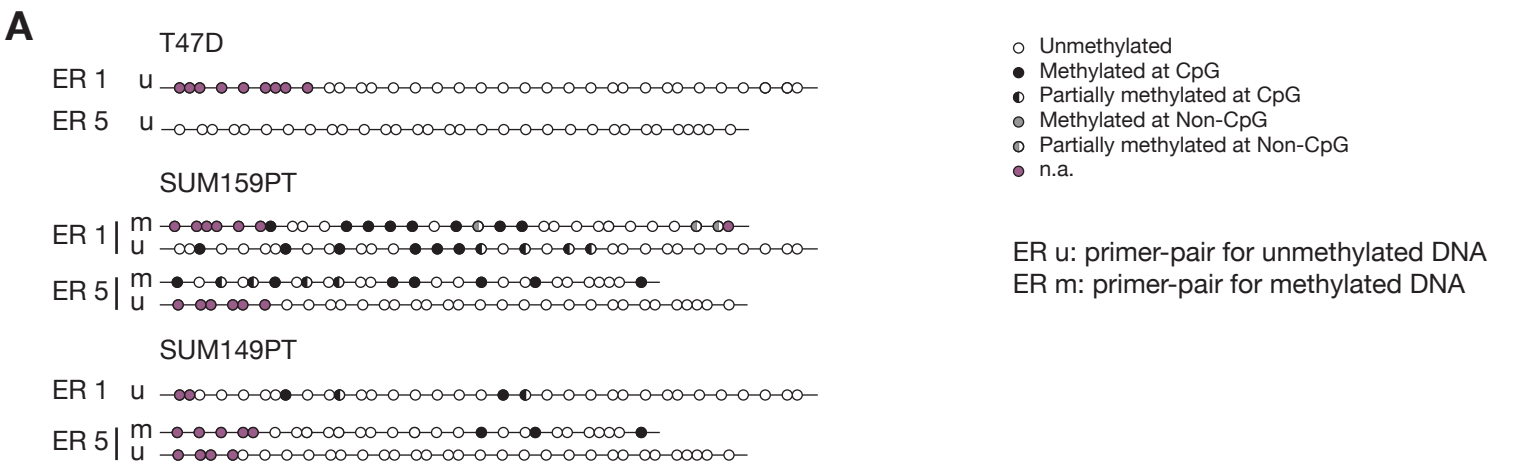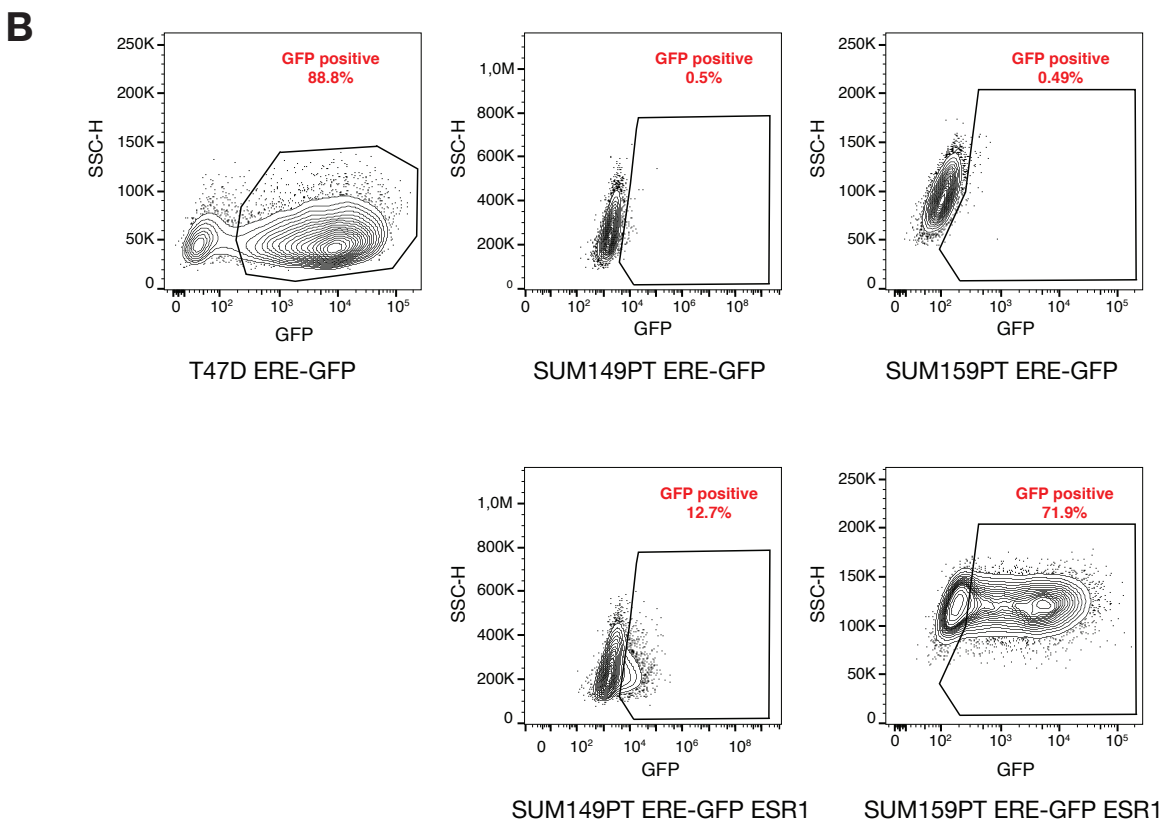

Supplement: Supplementary file 2 — Supplementary figure 1 [file 41388_2022_2429_MOESM2_ESM.pdf]

Figure S2

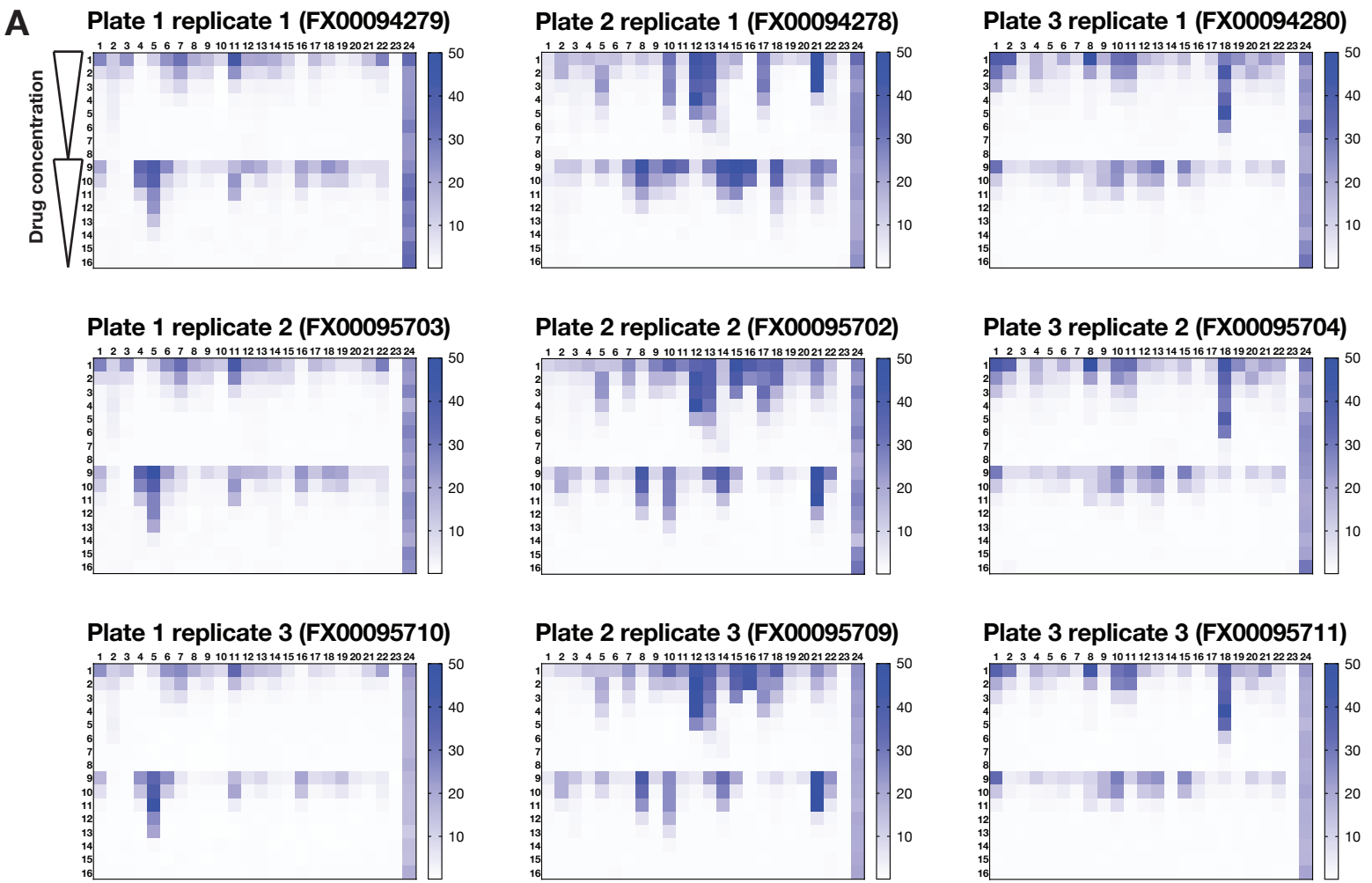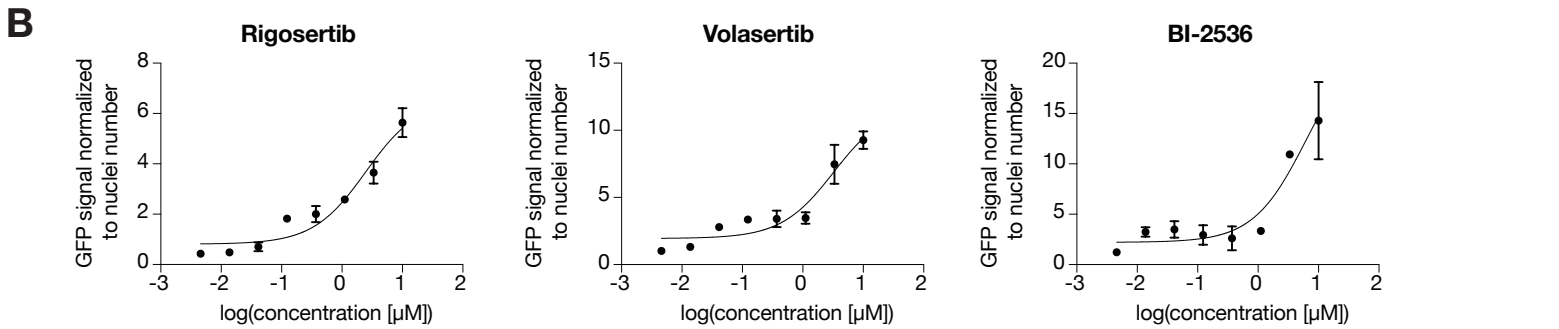

Supplement: Supplementary file 3 — Supplementary figure 2 [file 41388_2022_2429_MOESM3_ESM.pdf]

**Figure S3**

**A**

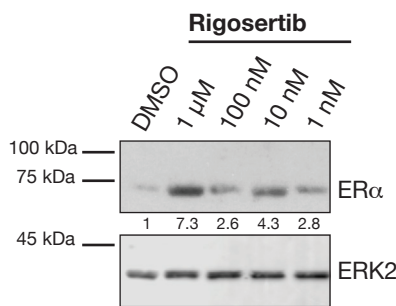

**B**

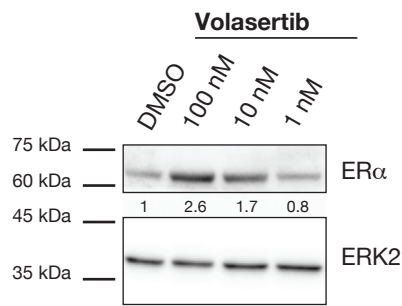

**C**

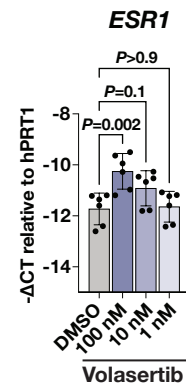

**D**

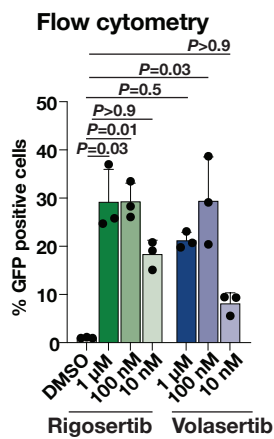

**E**

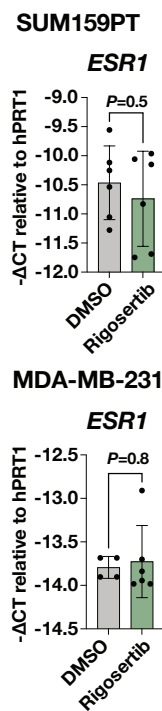

**F**

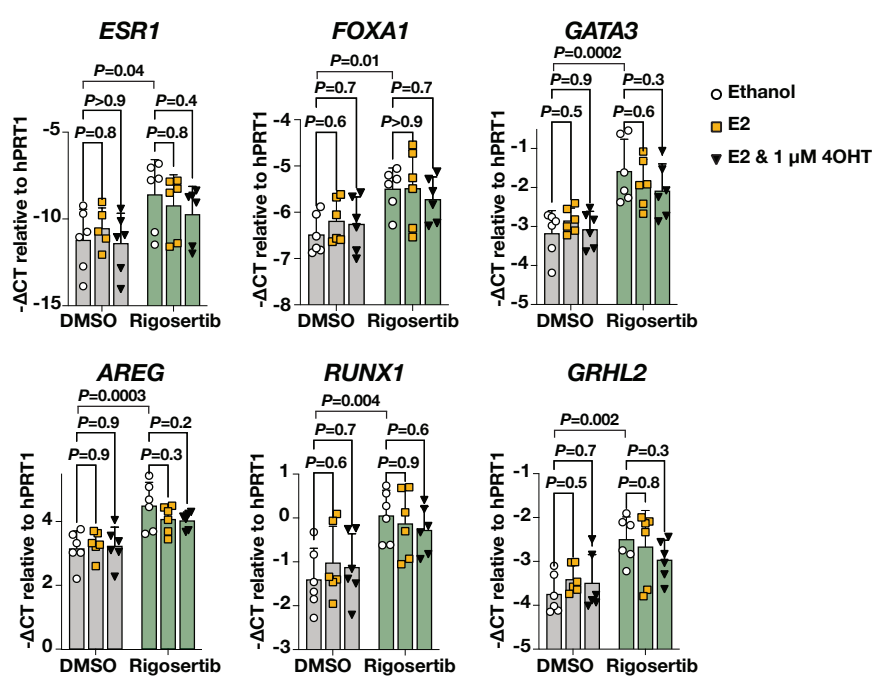

**G**

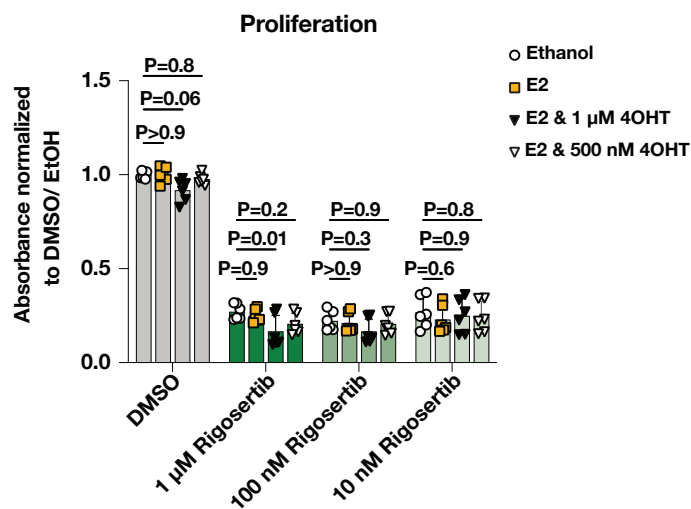

**H**

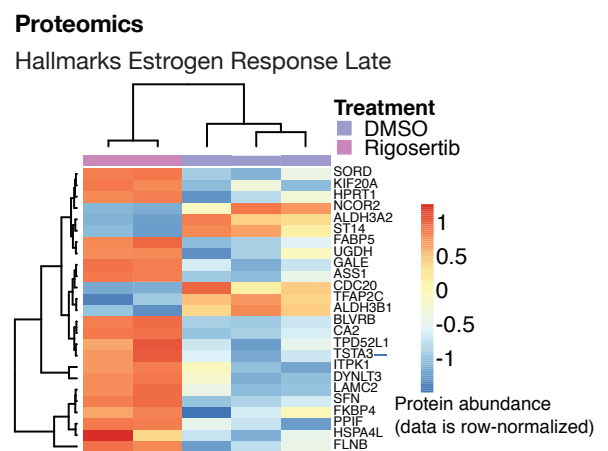

Supplement: Supplementary file 4 — Supplementary figure 3 [file 41388_2022_2429_MOESM4_ESM.pdf]

Figure S4

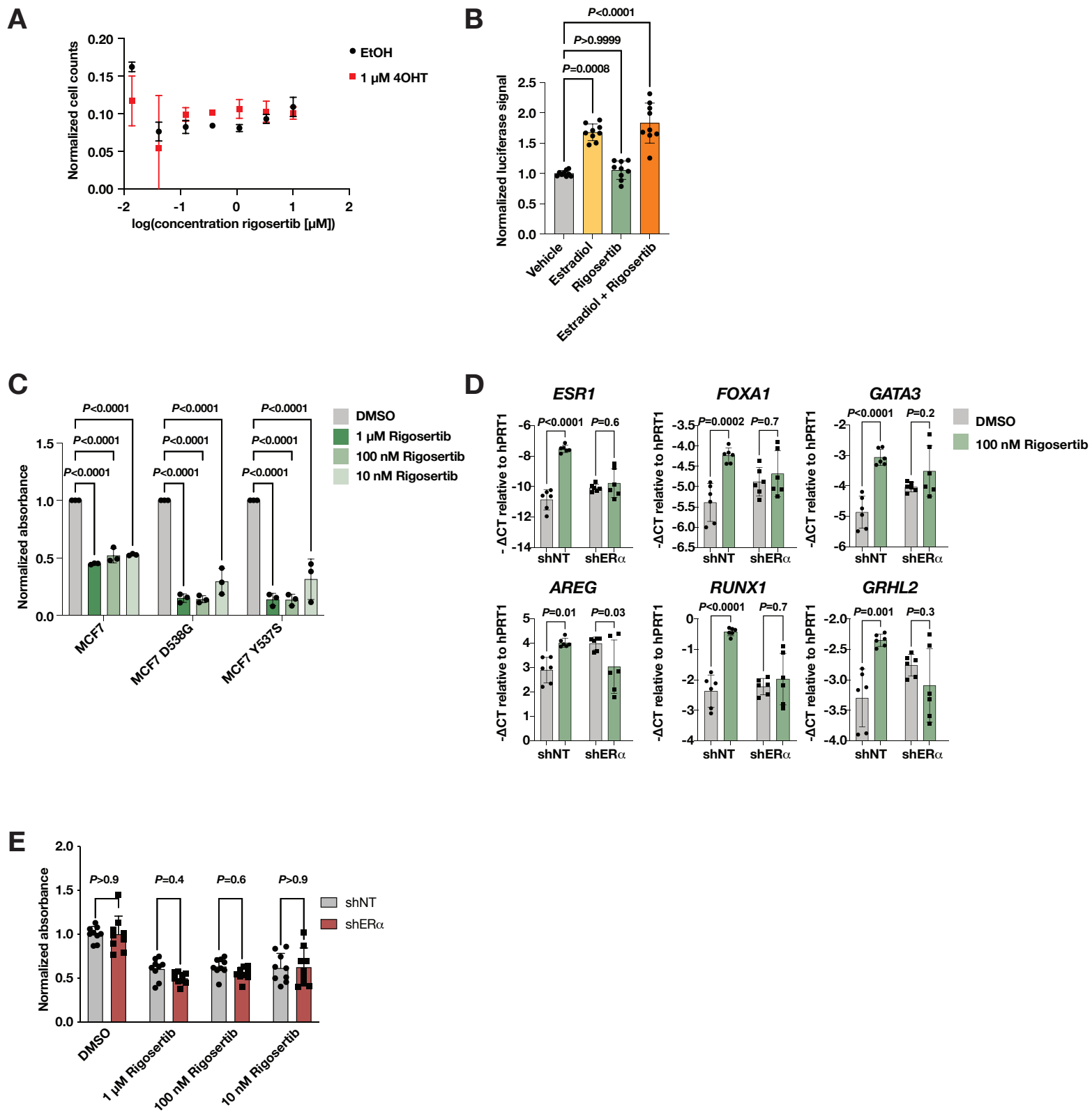

Supplement: Supplementary file 5 — Supplementary figure 4 [file 41388_2022_2429_MOESM5_ESM.pdf]

Figure S5

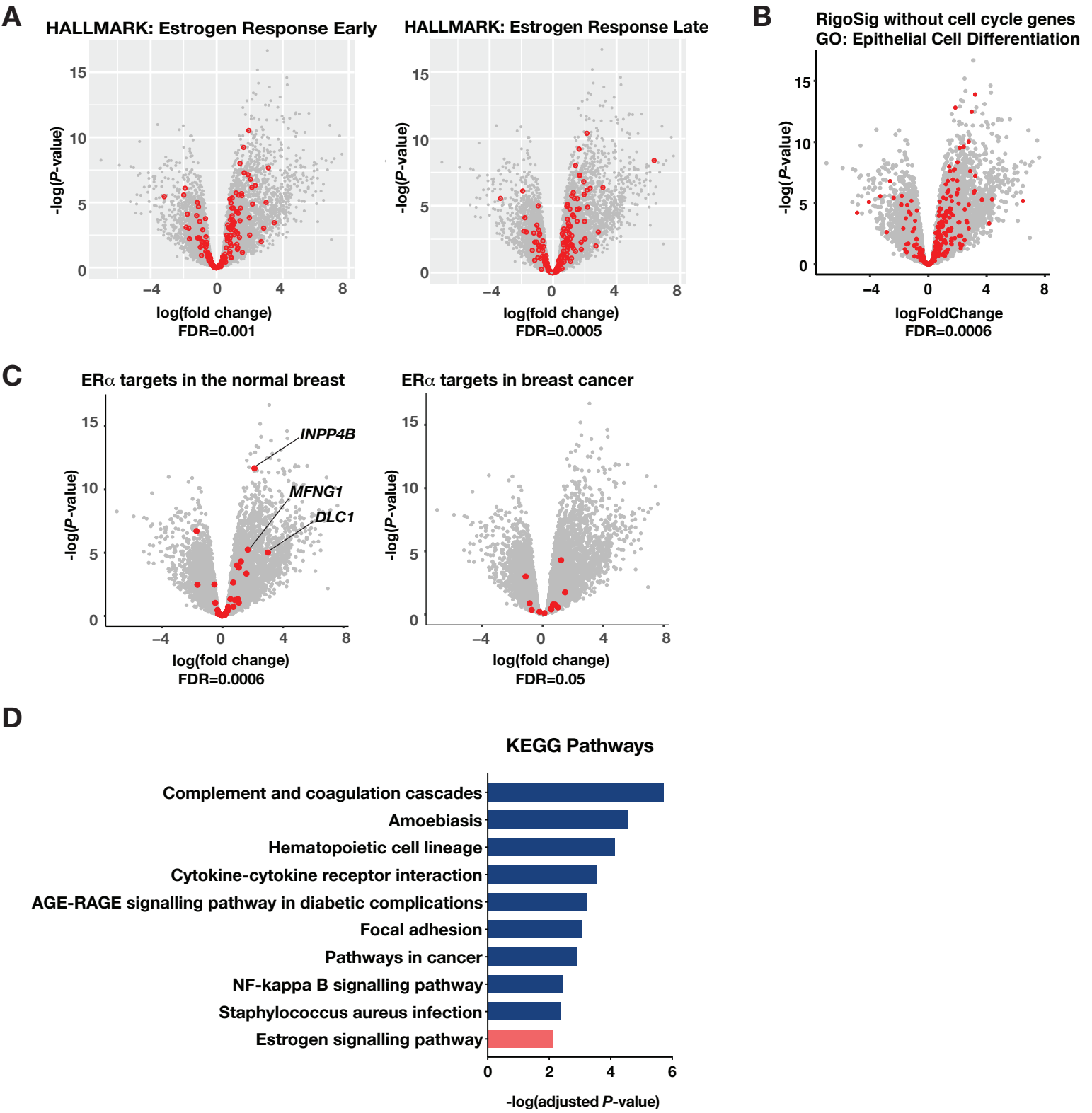

Supplement: Supplementary file 6 — Supplementary figure 5 [file 41388_2022_2429_MOESM6_ESM.pdf]

Figure S6

SUM149PT

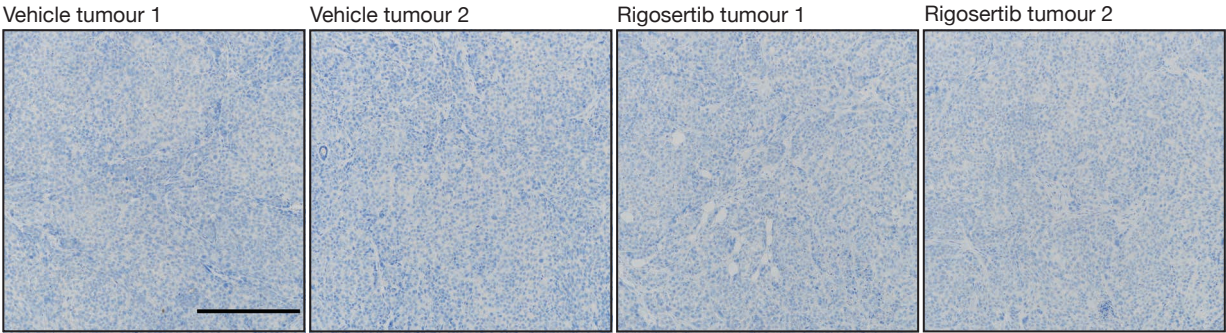

ERα

PDX1

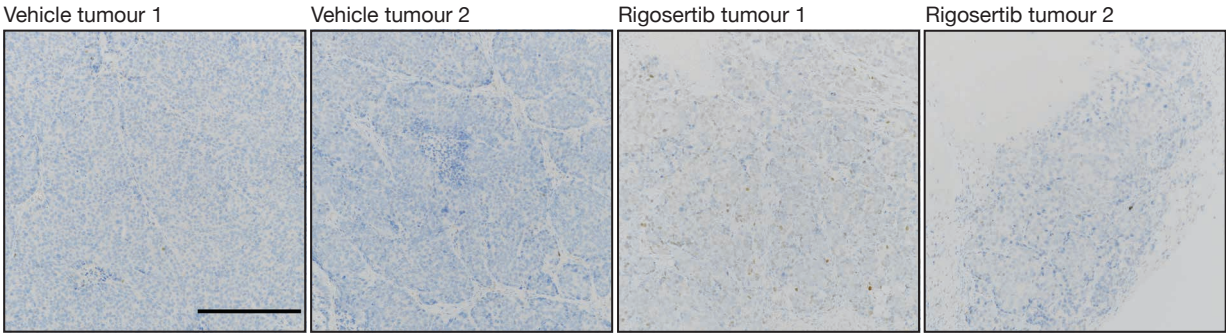

ERα

ERα positive tumour

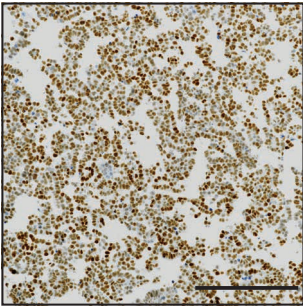

ERα

Supplement: Supplementary file 7 — Supplementary figure 6 [file 41388_2022_2429_MOESM7_ESM.pdf]
